# Genome-wide analysis of the gene families of resistance gene analogues in cotton and their response to Verticillium wilt

**DOI:** 10.1186/s12870-015-0508-3

**Published:** 2015-06-19

**Authors:** Jie-Yin Chen, Jin-Qun Huang, Nan-Yang Li, Xue-Feng Ma, Jin-Long Wang, Chuan Liu, Yong-Feng Liu, Yong Liang, Yu-Ming Bao, Xiao-Feng Dai

**Affiliations:** Laboratory of Cotton Disease, Institute of Agro-Products Processing Science & Technology, Chinese Academy of Agricultural Sciences, Beijing, 100193 China; BGI-Shenzhen, Shenzhen, Guangdong, 518083 China

**Keywords:** Cotton, Verticillium wilt-resistant, Resistance gene analogues, RGA-gene-rich clusters, *Verticillium dahliae* response loci

## Abstract

**Background:**

*Gossypium raimondii* is a Verticillium wilt-resistant cotton species whose genome encodes numerous disease resistance genes that play important roles in the defence against pathogens. However, the characteristics of resistance gene analogues (RGAs) and *Verticillium dahliae* response loci (VdRLs) have not been investigated on a global scale. In this study, the characteristics of RGA genes were systematically analysed using bioinformatics-driven methods. Moreover, the potential VdRLs involved in the defence response to Verticillium wilt were identified by RNA-seq and correlations with known resistance QTLs.

**Results:**

The *G. raimondii* genome encodes 1004 RGA genes, and most of these genes cluster in homology groups based on high levels of similarity. Interestingly, nearly half of the RGA genes occurred in 26 RGA-gene-rich clusters (Rgrcs). The homology analysis showed that sequence exchanges and tandem duplications frequently occurred within Rgrcs, and segmental duplications took place among the different Rgrcs. An RNA-seq analysis showed that the RGA genes play roles in cotton defence responses, forming 26 VdRLs inside in the Rgrcs after being inoculated with *V. dahliae*. A correlation analysis found that 12 VdRLs were adjacent to the known Verticillium wilt resistance QTLs, and that 5 were rich in NB-ARC domain-containing disease resistance genes.

**Conclusions:**

The cotton genome contains numerous RGA genes, and nearly half of them are located in clusters, which evolved by sequence exchanges, tandem duplications and segmental duplications. In the Rgrcs, 26 loci were induced by the *V. dahliae* inoculation, and 12 are in the vicinity of known Verticillium wilt resistance QTLs.

**Electronic supplementary material:**

The online version of this article (doi:10.1186/s12870-015-0508-3) contains supplementary material, which is available to authorized users.

## Background

Resistance (*R*) genes play a central role in recognising effectors from pathogens and in triggering downstream signalling during plant disease resistance [1, 2]. To date, more than 112 *R* genes and 104,310 putative *R*-genes present in a wide variety of plants species and conferring resistance to 122 pathogens [3]. The known R proteins can be grouped into several super-families based on the presence of a few structural motifs, including nucleotide-binding sites (NBSs), leucine-rich repeat (LRR) domains, Toll/Interleukin-1 receptor (TIR) domains, coiled-coil (CC) domains and transmembrane (TM) regions [4, 5]. Generally, the most prevalent *R* genes in plants are of the NBS-LRR type, which are divided into two sub-classes based on the presence of an N-terminal CC or TIR domain [6, 7]. For example, 480 NBS-LRR proteins are encoded by the rice genome [8].

Previous studies demonstrated that many *R* genes are clustered in plant genomes [9]. To date, clusters of *R* genes have been reported in several plant genomes, including *Arabidopsis* [7], rice [10], soybean [11], *Lotus japonicus* [12], *Medicago truncatula* [13] and *Phaseolus vulgaris* [14]. In *Arabidopsis*, the genome was found to encode 159 NBS-LRR genes, and 113 of these genes occurred in 38 clusters [15]. A similar phenomenon was also found in the rice genome, in which 76 % of the rice NBS-LRR genes was arranged in 44 gene clusters, with the others occurring as singletons [8]. The lengths of RGA gene clusters varied from dozens of kilobases (kb) to several megabases (Mb). For example, RGA genes were tightly linked to the *RPP5* cluster in *Arabidopsis*, which covers less than 100 kb [16], while the RGA genes were distributed over several Mb of the *RGC2* locus in lettuce [17]. Different *R* genes from the same cluster can confer resistance to different pathogens or to different variants of a single pathogen [18, 19]. For example, the Cf*-9* gene cluster contains two Cf*-9* and Cf*-9B* homologues that recognise the Avr9 and Avr9B effectors, respectively, in *Cladosporium fulvum*, and contribute to the resistance against tomato leaf mould disease. Other homologous genes in the cluster may serve as a reservoir of variation for the generation of *R* genes with new specificities [20–22].

Previous research suggested that the evolution of RGA clusters is usually mediated by sequence exchange, tandem duplication, segmental duplication, or gene conversion [9, 23, 24]. Frequent sequence exchanges tend to homogenize the members of a gene family, like the *RGC2* genes in lettuce [25], the *R1* cluster in *Solanum demissum*, and the Cf*-9* cluster in tomato [26, 27]. Tandem and segmental genomic duplications are also important in the evolution of RGA genes [23], which frequently occur in NBS-LRR genes clusters, and led to the formation of the phylogenetic lineage of NBS-LRR genes in the *Arabidopsis* genome [7, 28]. The evolution of the *HcrVf* cluster in apple was primarily dependent on gene duplication, with four *HcrVf* genes originating from a single progenitor gene by two sequential duplication events [29]. RGA’s evolution by gene conversion resulted in high levels of sequence similarity, close physical clustering, and the local recombination rate [15, 28, 30]. In conclusion, the plants employed a complicated mechanism on the RGA genes evolution to response the variations of pathogens.

Cotton is an important crop worldwide because of its natural fibres and oil seeds. The cotton acreage in China has reached 4.69 million hectares, which produced 6.83 million tons of cotton in 2012 (Data from the National Bureau of Statistics in China). At present, Verticillium wilt caused by *Verticillium dahliae* is the most destructive disease of cotton, and the survival structures produced by pathogens may remain viable in the soil, persistently threatening crops, for more than 20 years [31]. In some years, more than 50 % of the cotton acreage is affected by Verticillium wilt, significantly reducing the fibre quality and resulting in yield losses (National Cotton Council of America Disease Database). Because of its unique ecological niche in the plant’s vascular, Verticillium wilt is difficult to control using fungicides, chemicals and cultivation measures [32]. Improving genetic resistance is considered the best method to overcome Verticillium wilt, and at least 80 different Verticillium wilt resistance quantitative trait loci (QTLs) have been reported in cotton [33–37]. However, *Gossypium hirsutum* appears to lack genetic resistance against *V. dahliae* [38, 39].

*Gossypium barbadense*, which is a cultivated tetraploid cotton species, showed resistance or tolerance to Verticillium wilt [40]. To date, the transcriptomes and proteomes of this Verticillium wilt-resistant cotton’s responses to *V. dahliae* have been analysed, and phytoalexin biosynthesis and hormone signalling were found to have important roles in pathogen defense [41–46]. Moreover, several genes that contribute to the defence response against Verticillium wilt have been reported, including *GbCAD1*, *GbSSI2* [43], *GbRLK* [47], *GbSTK* [48], *GbTLP1* [49] and *GbVe*/*GbVe1* [50, 51].

Recently, the genome sequence of a diploid cotton, *Gossypium raimondii*, which is a Verticillium wilt-resistant wild relative of cotton, was completed [52, 53]. It is commonly thought that the tetraploid cotton species *G. hirsutum* and *G. barbadense* were derived from a cross between a D-genome species as the pollen-providing parent and an A-genome species as the maternal parent, and that *G. raimondii* is the putative D-genome parent [54, 55]. Previous research showed that the cotton genome encodes numerous NBS domains and that some of these genes formed gene clusters [53, 56]. A transcriptome analysis showed that some RGAs are involved in the defence response against *V. dahliae* [42, 46]. However, there are no systematic studies of RGA genes in the cotton genome, and the genetic resistance to Verticillium wilt is unclear.

In this study, a global analysis, including sequence features, gene distribution and the evolution of RGA genes in the *G. raimondii* genome was performed. High-throughput RNA-seq was used to identify the RGA genes’ transcriptome in a *V. dahlia*-resistant cultivar of *G. barbadense* and to screen for potential *Verticillium dahliae* response loci (VdRLs) in the gene clusters. Moreover, the association between the VdRLs and Verticillium wilt resistance QTLs were analysed to screen the Verticillium wilt-response loci in cotton.

## Results

### Analysis of RGA genes in the *G. raimondii* genome

In this study, we focused on the RGA genes in the *G. ramondii* genome that probably participate in the disease resistance response. In total, 1004 RGA genes were classified into 11 families (R-I – R-XI) based on the integrated annotation of conserved motifs or domains in the *G. ramondii* genome [53]. The genome included 32 CC-NBS-LRR genes, 60 cysteine-rich receptor-like kinase (RLK) genes, 46 genes encoding disease resistance family proteins/LRR family proteins, 58 genes encoding leucine-rich receptor-like protein kinase family proteins, 225 genes encoding LRR protein kinase family proteins, 44 genes encoding LRR receptor-like protein kinase family proteins, 78 genes encoding LRR transmembrane protein kinases, 79 genes encoding LRR and NB-ARC (Nucleotide-Binding adaptor shared by APAF-1, Resistance proteins and CED-4) domain-containing disease resistance proteins, 194 genes encoding NB-ARC domain-containing disease resistance proteins, 144 receptor-like proteins (RLP) genes and 44 TIR-NBS-LRR genes (Additional file 1: Table S1). A statistical analysis showed that more than half of the RGA genes were located on three chromosomes, with 194, 182 and 143 on Chr09, Chr07 and Chr11, respectively (Additional file 2: Figure S1). These results indicated that the cotton genome contains many RGA genes and numerous of them trend to enrich in several chromosome in cotton genome.

**Fig. 1:**
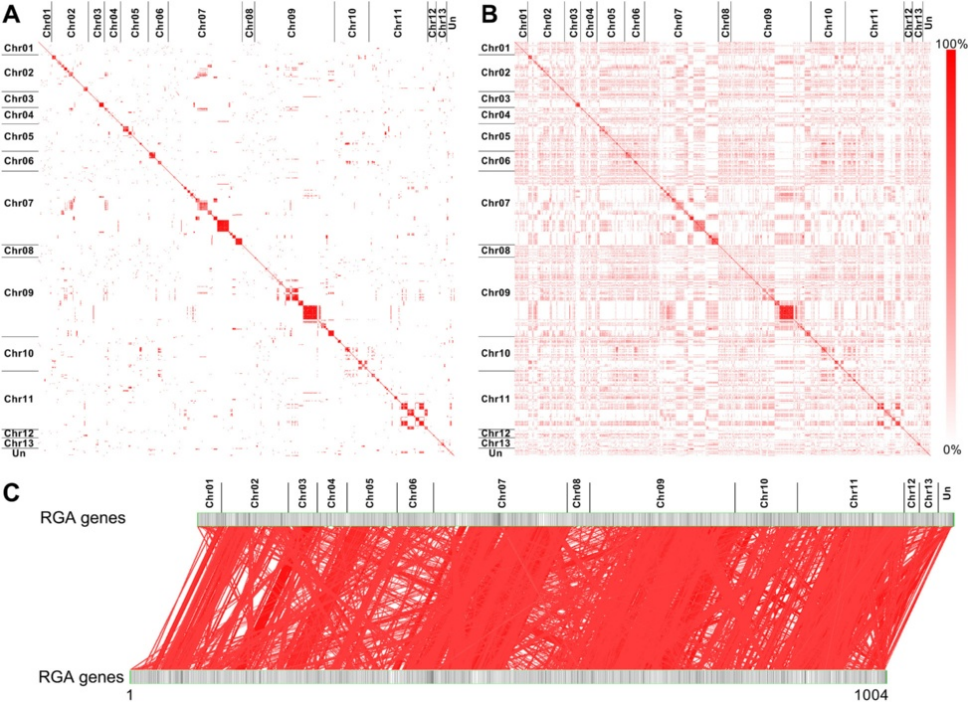
Similarity analysis of RGA genes in the *G. raimondii* genome. (**A**) The identity matrix of all RGA genes versus all RGA genes. The RGA genes were arranged in a series from Chr01 to Chr13. “UN” represents the RGA genes that cannot presently be mapped to chromosomes. The identity level between each two genes was determined by BLASTN (Version 2.2.23). (**B**) The identity matrix of all RGAs encoding proteins versus all RGAs encoding proteins. The identity level between each two proteins was determined using the BLASTP program (Version 2.2.23). (**C**) Homology analysis between two chimeric sequences of RGA genes. The chimeric sequence was constructed by ligating the RGA sequences in a series from Chr01 to Chr13. The similarity blocks were determined using the BLASTN program (Version 2.2.23) with chimeric sequences, ignoring self-matches and filtering out the similarity blocks based on the length of the smallest RGA gene (216 bp)

Generally, RGA genes contain conserved domains or motifs, such as NBSs and LRRs. In a comparative analysis, most of the RGA genes, and their encoded proteins, showed a high identity with one another (Fig. 1A, B), particularly RGA genes on Chr07 and Chr09, which shared high identities (up to 80 %) with one another (Additional file 1: Table S2). To investigate the correlation among all RGA genes, the similarity among RGA genes were compared according to the chimeric sequence which connected the RGA gene sequences from Chr01 to Chr13 in a series. Interestingly, the comparison of the chimeric sequence with itself showed a high similarity apart from small similarity blocks (less than the length of the smallest RGA gene, 216 bp) and self-match (Fig. 1C), indicating that many RGA genes are similar in the cotton genome. Moreover, the chimeric sequence segments from the same chromosome were more similar than sequence segments from different chromosomes (Fig. 1C), indicating that RGA genes on the same chromosome were more closely related than genes on different chromosomes.

The homology clustering of RGA genes also indicated that RGA genes are conserved in cotton. Of the 1004 RGA genes, 974 could be divided into 45 homology groups (HG), with at least two genes in each HG, under the clustering conditions of match rate and identity being more than 33 % and 30 %, respectively. Of these, 838 were classified into 11 HGs, with HG13 containing the minimum 23 genes and HG17 containing the maximum 242 genes (Additional file 1: Table S3). Not surprisingly, most RGA genes in the same family could be clustered into a single HG based on a conserved feature. For example, five-sixths of the RGA genes in the R-II family were clustered into HG22. However, the genes of five RGA gene families were clustered into multiple groups, including R-I, R-V, R-VIII and R-IX. The RGA genes of the R-V family were clustered into two major HGs, HG17 and HG21 (Additional file 1: Table S3), indicating that the RGA gene families were not always clustered in one HG but could be clustered into different HGs. Moreover, the RGA genes could also be clustered into HGs using highly rigorous conditions. The 306 RGA genes were divided into 104 HGs when the match rate and identity were more than 80 % for each gene (Additional file 2: Figure S2). The RGA genes in the same HGs are physically linked, such as 7 genes in the sub-HG of HG05 (HG05-04) that are closely linked in a small region that encodes 11 genes (Gorai.007G324100.1–Gorai.007G325100.1) (Additional file 1: Table S4). These results suggested that many RGA genes, which are probably multi-copy genes in cotton, are closely linked in the cotton genome.

**Fig. 2:**
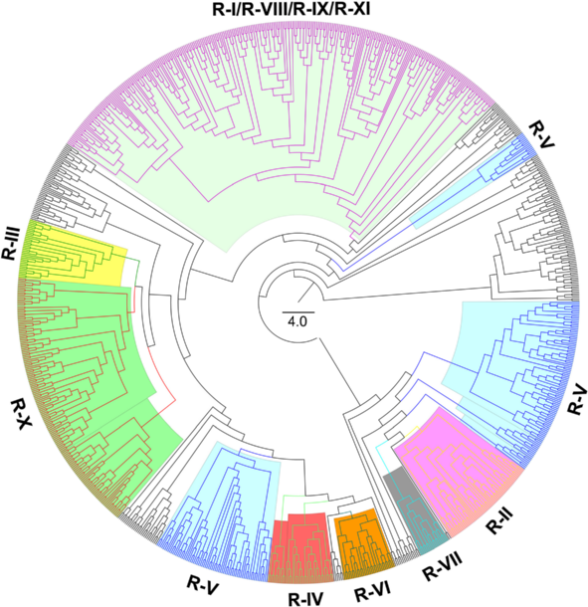
Phylogeny analyses of RGA genes in the *G. raimondii* genome. The phylogenetic tree of RGA genes was constructed using the protein sequences by the neighbour-joining method, with 1000 bootstrap replicates. The branches of the mixed clade included four RGA gene families, which are marked in purple. Other conserved clades of RGA gene families are rendered in different colours

The phylogenetic relationship analysis of RGA genes showed that most RGA genes could be arranged in clades in accordance with RGA gene families, such as R-II, R-III and R-IV (Fig. 2). These results also corresponded to the homology clustering, showing that the major HGs in an RGA gene family were arranged in a clade. For example, most R-II family genes were clustered into HG22, which was arranged in a single clade (Fig. 2; Additional file 1: Table S3). Although most of the R-V family genes could be arranged together in the phylogenetic tree, the R-V clade was split into three parts (Fig. 2), which indicated that variation occurred in the R-V family. More persuasive evidence showed four RGA gene families (R-I, R-VIII, R-IX and R-XI) which mainly contain the NBSs and LRRs domain were arranged in a mixed clade (Fig. 2). Together, these results indicated that the variation in RGA genes is as important as the conservation during the cotton genome’s evolution.

### Many RGA genes are deposited in gene clusters

**Fig. 3:**
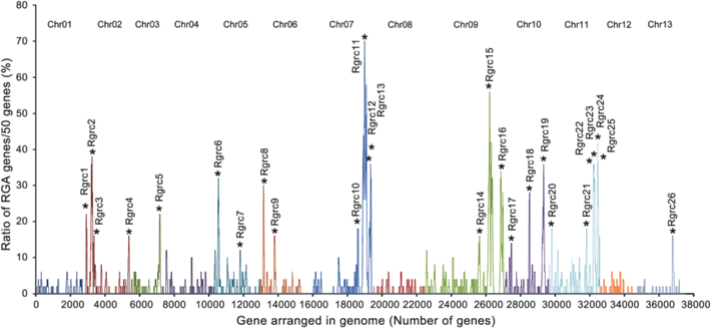
The distribution of Rgrcs in the *G. raimondii* genome. All genes encoded by the *G. raimondii* genome were arranged in a series from Chr01 to Chr13. The ratio of RGA genes was calculated in the moving window (50 genes/window, walking forward 10 genes each time). RGA gene frequencies greater than 10 % were considered Rgrcs and clusters only containing 6 RGA genes in a window, but distributed evenly, were removed. The X-axis represents the number of genes in the cotton genome and the Y-axis represents the RGA gene ratio in the moving window

In the *G. ramondii* genome, nearly half of the RGA genes were allocated to 26 Rgrcs (Fig. 3; Additional file 2: Figure S3). The total length of these Rgrcs is ~16.7 Mb, and there were 1148 genes, including 489 RGA genes. The average proportion of RGA genes in Rgrcs is significantly higher than in the whole genome, 42.6 % compared with 2.7 %. The average whole gene density was higher in Rgrcs (14.5 kb/gene) than in the whole genome (19.7 kb/gene) (Additional file 1: Table S5). Among these Rgrcs, Rgrc14 and Rgrc11 are the two largest clusters, which cover ~4.2 and 3.3 Mb, respectively, and contained 82 and 103 RGA genes, respectively (Additional file 1: Table S5). Most of the Rgrcs were located on Chr02, Chr07, Chr09, Chr10 and Chr11 (Fig. 3; Additional file 1: Table S5). Moreover, more than half of the RGA genes in the eight gene families occurred in these clusters, except those of RGA families R-IV, R-V and R-VII. Only 15.5 % of RGA genes in the R-V family occurred in Rgrc clusters (Additional file 1: Table S6). These results suggested that many RGA genes occur in gene clusters in the cotton genome.

**Fig. 4:**
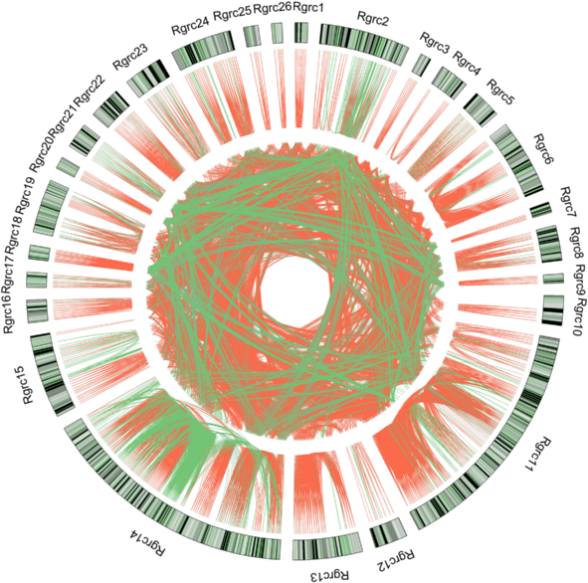
Homology clustering of proteins encoded by genes in the Rgrcs of the *G. raimondii* genome. The homologous relationships were determined among proteins encoded by genes in the Rgrcs. The same homology groups of RGA genes are linked with red lines, while other genes in the same homology groups are linked with green lines. The outer ring represents the homology groups inside in Rgrcs, and the inner ring represents homology groups in different Rgrcs

To investigate how Rgrcs are related, all of the proteins encoded by Rgrcs were analysed using homology clustering. Clearly, most RGA genes are homologous to those clustered in the same HGs within the Rgrcs. This is also true for other genes in the Rgrcs that do not encode RGA genes, such as Rgrc2, Rgrc14 and Rgrc15. (Fig. 4). The homology of most genes within Rgrcs probably indicates that Rgrcs undergo tandem duplications or sequence exchanges during their evolution. Moreover, most proteins encoded in different Rgrcs also clustered into same HGs (Fig. 4). Thus, the genes in different Rgrcs are homologous, indicating that some Rgrcs were probably generated from other Rgrcs by segmental duplications in cotton.

Homology analysis of the chimeric sequence, all the Rgrcs sequences connected in series from Chr01 to Chr13, showed that the Rgrcs was highly similar after apart from the small (less than the length of the smallest RGA gene, 216 bp) and self-matching similarity blocks (Additional file 2: Figure S4A). In total, 984 high similarity blocks in the chimeric sequence were matched to each other (up to 3 kb, ignoring self-match), except for the sequences of Rgrc4 and Rgrc20, and the identities of almost all the similarity blocks were close to 80 % (Additional file 2: Figure S4B/C). Of the similarity blocks, 589 belonged to “Rgrc-self-similarity”, including 300 blocks within Rgrc14, and 78 blocks inside in Rgrc11 (Additional file 2: Figure S4B), indicating that the Rgrc sequences are similar by themselves, which could be the result of tandem duplication or sequence exchange. However, parts of the similarity blocks were also found among different Rgrcs, such as 42 matching blocks between Rgrc11 and Rgrc14, and 22 matching blocks between Rgrc11 and Rgrc24. (Additional file 2: Figure S4B), suggesting that some Rgrcs originated by segmental duplication in cotton.

### RGA gene expression responses to *V. dahliae* infection

#### Analysis of RNA-seq data

In this study, *G. barbadense* cv. 7124, which is considered to be *V. dahliae*-resistant (Additional file 2: Figure S5), was inoculated with the highly aggressive defoliating *V. dahliae* strain Vd991. The inoculated root samples (2, 6, 12, 24, 48 and 72 h) were collected to identify differentially expressed genes (DEGs) of RGAs using high-throughput RNA-seq. For extremely deep sequencing, ~200 million clean reads for each sample were generated, with quality control (Q ≥ 20) (Additional file 1: Table S7). Of these reads, ~76 % matched the reference genome of *G. raimondii*, including ~140 million unique matched reads and ~13 million multi-position matched reads (Additional file 1: Table S7).

For DEG detection, the reads per exon kb per million mapped sequence reads (RPKM) was calculated for each gene and filtered using the false discovery rate (FDR) and with the *p*-value. In total, 28,360 DEGs were detected in the cotton genome at six inoculated time points, with 13,229 genes in common at different time points (FDR < 0.001, *p* < 0.001), 17,517 DEGs in all inoculated time points and 9811 genes in common (FDR < 0.001, *p* < 0.001, and log_2_Ratio ≥ |1.0|), 8122 DEGs in all inoculated time points and 5106 genes in common (FDR < 0.001, *p* < 0.001, and log_2_Ratio ≥ |2.0|) (Additional file 1: Table S8; Additional file 3: Table S9). The number of up-regulated DEGs peaked at 48 h after inoculation, and the number of down-regulated DEGs gradually decreased from 2 to 72 h (Additional file 2: Figure S6), which corresponded to the important infection time point of 48 h in *V. dahliae*, for the penetration of hyphae into the roots was evident about two days [57–60].

#### DEGs of RGA genes

In the DEGs set, 723 RGA genes were induced in cotton inoculated with *V. dahliae*, with 319 RGA genes in common at six time points (FDR < 0.001, *p* < 0.001) (Additional file 1: Table S8). Real-time quantitative RT-PCR (qRT-PCR) showed that the fold-change of DEGs is reliable (Additional file 2: Figure S7). As with the DEGs in the whole genome, the DEGs of RGA genes were also obviously induced at 48 h after inoculation (Additional file 2: Figure S6). The statistical analysis of DEGs showed that all 11 RGA families could respond to the *V. dahliae* inoculation at all of the time points, although the proportion of DEGs in the RLP family was relatively small (Additional file 1: Table S10). These results suggested that RGA genes are involved in the cotton response to *V. dahliae*. The expression pattern analysis showed that RGA gene families that responded to *V. dahliae* could be classified into the early response stage (~2–12 h) and later response stage (~24–72 h). In the later response stage, the number of RGA genes and their expression levels were induced more obvious than in the early response stage (Additional file 2: Figure S8). These results indicated that activating the later response stage is important to the resistant cotton plant’s response to *V. dahliae*.

**Fig. 5:**
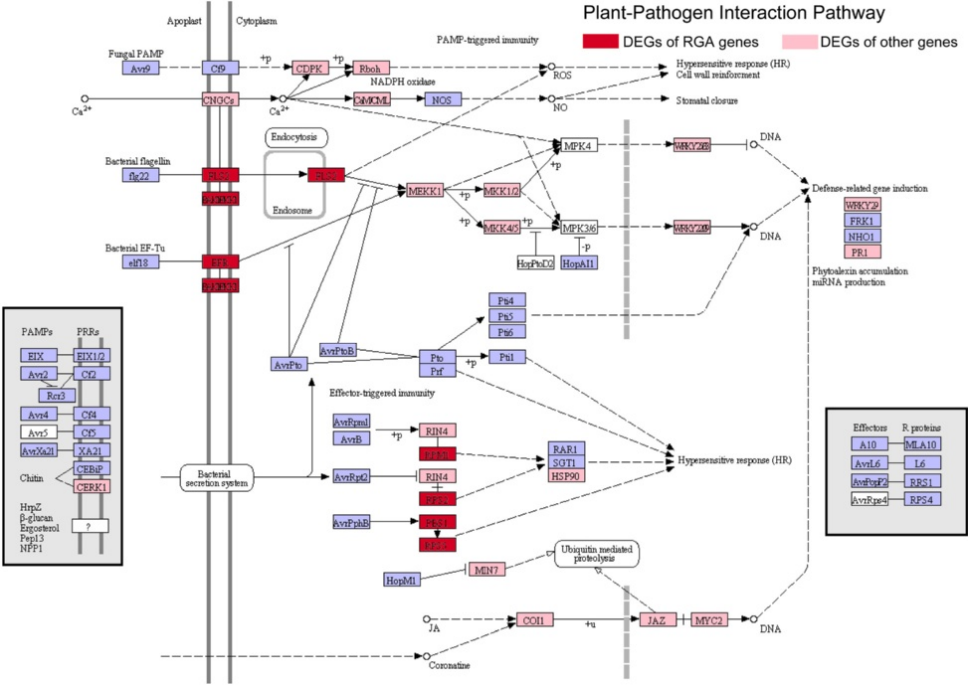
DEGs homologous to the genes of the plant-pathogen interaction pathway. The DEG genes were screened using FDR < 0.001, *p* < 0.001, and log_2_Ratio ≥ |1.0| at all six inoculation time points. The red box represents the differentially expressed RGA genes that map to the plant-pathogen interaction pathway, the pink box represents the other DEGs that map to the plant-pathogen interaction pathway, and the blue and white box represents the reference KEGG pathway (map04626)

Many genes in the plant-pathogen interaction pathway are RGA genes, which play an important role in disease resistance. In this study, 451 differentially expressed RGA genes were induced in cotton inoculated with *V. dahliae*, and mapped to the plant-pathogen interaction pathway based on the Kyoto Encyclopedia of Genes and Genomes (KEGG) annotation (Fig. 5), including eight types of homologous genes, such as BAK1, FLS2 and EFR (Additional file 1: Table S11). Moreover, some genes homologous to signal factors in the plant-pathogen interaction pathway, which are not RGA genes, were also activated, such as protein kinases and transcription factors (Fig. 5). In addition, genes in the phytoalexin biosynthesis pathways, including those for phenylpropanoids, flavonoids and diterpenoids, were also induced in cotton in response to *V. dahliae* (Additional file 2: Figure S9). Overall, the transcriptome results indicated that many RGA genes, which probably participated in the plant-pathogen interaction pathway and regulated the defence response, were induced in cotton.

#### DEGs in Rgrcs

**Fig. 6:**
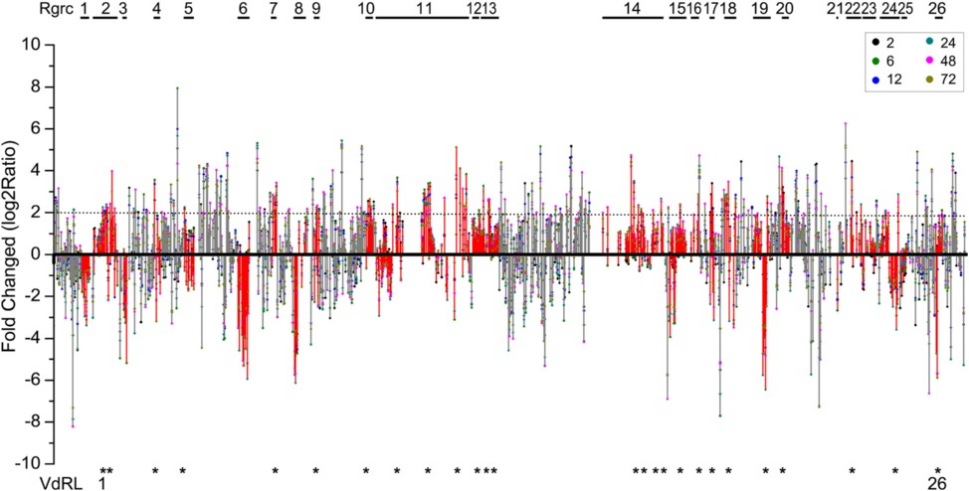
Analysis of RGA gene expression patterns and the screening of potential VdRLs. The RGA genes were arranged in a series from Chr01 to Chr13. RGA genes belonging to the 26 Rgrcs are shown in red. The fold-change of log_2_Ratio ≥ |2.0| is marked in dotted lines. The potential VdRLs were screened from Rgrcs using a log_2_Ratio ≥ |2.0|, and having more than one infection time point up-regulated. The potential VdRLs were marked with asterisks. The numbers 2, 6, 12, 24, 48, and 72 in the boxes represent the time points (in hours) of the cotton inoculation with *V. dahliae*

The expression pattern analysis of DEGs in Rgrcs indicated that the RGA genes were up-regulated more often than other genes in Rgrcs (Additional file 2: Figure S10), which suggested that RGA genes were more sensitive to *V. dahliae* inoculation than the other genes in Rgrcs. To investigate the potential RGA gene responses to *V. dahliae* infection, highly rigorous conditions (log_2_Ratio ≥ |2.0|, with more than one up-regulated post-infection time point) were used for screening in this study. In total, 168 differentially expressed RGA genes were identified as potential Verticillium wilt response genes. Of these genes, the proportion of potential Verticillium wilt resistance genes in R-II, R-III and R-IV families was higher than in other families (Additional file 1: Table S12 and Table S13). Notably, 64 DEGs occurred in 19 Rgrcs, and 63 of them were distributed in the 26 small regions defined VdRL01 to VdRL26 (Fig. 6; Additional file 1: Table S12-S14). The total length of the VdRLs is ~2.4 Mb, and a minimum of 15 VdRLs contain at least two significantly differentially expressed RGA genes (Additional file 1: Table S14). A total of 39 differentially expressed RGA genes in the VdRLs belonged to the R-II, R-VII and R-IX families (Additional file 1: Table S12), indicating that these RGA genes were important to the cotton response to Verticillium wilt. Moreover, most VdRLs were primarily distributed in the small regions of a few chromosomes, particularly Chr07 and Chr09, which included seven and six VdRLs respectively (Additional file 1: Table S14). A further analysis showed that the RGA genes of nearly half of the VdRLs encoded NB-ARC domain-containing disease resistance proteins, and the RGA genes of the other VdRLs primarily encoded cysteine-rich RLKs, leucine-rich repeat protein kinase family proteins and RLPs (Additional file 1: Table S15). These results indicated that some RGA genes in the Rgrcs were strongly induced and a portion of them formed the VdRLs that participated in Verticillium wilt response in cotton.

### VdRLs adjacent to Verticillium wilt resistance QTLs

**Fig. 7:**
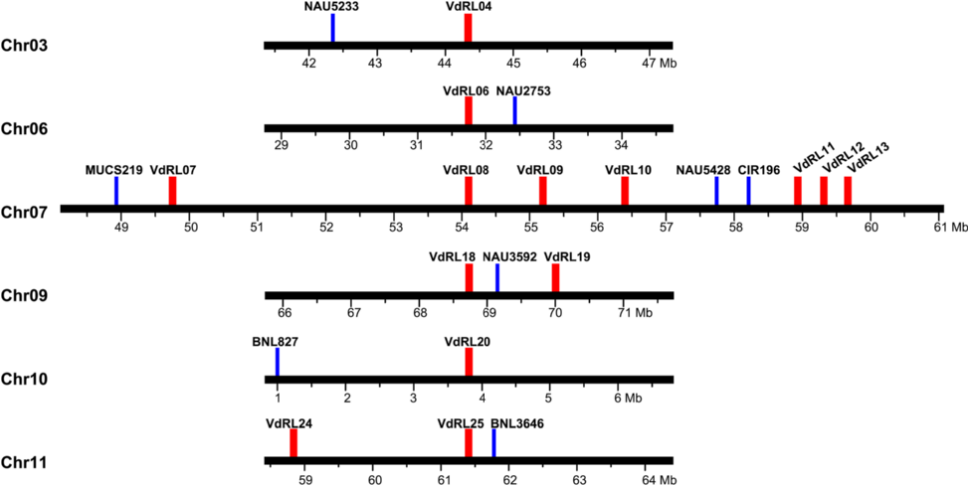
Correlation analysis between VdRLs and Verticillium wilt resistance QTLs in cotton. The physical location of the VdRLs and disease resistance QTLs were determined by their positions in the diploid cotton genome of *G. raimondii*. The VdRLs are marked in red and the QTLs markers are labelled in blue

To detect the co-localization of VdRLs and QTLs, which had been identified to be associated with the Verticillium wilt resistance in cotton [33–37], the locations of these QTLs in the diploid cotton genome were analysed based on the information provided by their corresponding markers. Among the 81 markers for these QTLs, 70 could be located on the diploid cotton genome (Additional file 1: Table S16), and 8 markers were adjacent to the VdRLs (Fig. 7; Additional file 1: Table S14). In total, 13 VdRLs were located on 6 chromosomes (3, 6, 7, 9, 10 and 11) with a physical distance of less than 3 Mb to the closest QTL marker, and 6 of them (VdRL06, VdRL07, VdRL11, VdRL18, VdRL19 and VdRL25) were less than 1 Mb from the closest marker (Fig. 7; Additional file 1: Table S14), suggesting that these VdRLs were positively correlated with the Verticillium wilt response. Moreover, the RGA genes in five VdRLs (VdRL07, VdRL11, VdRL12, VdRL13 and VdRL18) encoded NB-ARC domain-containing disease resistance proteins, of which three (VdRL07, VdRL11 and VdRL18) were close to Verticillium wilt resistance QTLs (Additional file 1: Table S14 and Additional file 1: Table S15).

Interestingly, six VdRLs (VdRL07 and VdRL09-VdRL13) located on Chr07 were found close to three Verticillium wilt resistance QTL markers (with a physical distance of less than 3 Mb), MUCS219, NAU5428 and CIR196 (Fig. 7; Additional file 1: Table S14). This region, in fact, extends about 10 Mb, which includes Rgrc10 and Rgrc11, and contains seven VdRLs (VdRL07-VdRL13). The physical distance betweenVdRL08 and the closest marker is ~3.66 Mb (Fig. 7; Additional file 1: Table S14). Of these seven VdRLs on Chr07, five were enriched for the NB-ARC domain-containing disease resistance genes, and two (VdRL07 and VdRL13) were close to the Verticillium wilt resistance QTLs (less than 1 Mb) (Fig. 7; Additional file 1: Table S14). Overall, these results suggested that the VdRLs located on Chr07, which mainly encoded NB-ARC domain-containing disease resistance proteins, were closely associated with Verticillium wilt resistance in cotton.

## Discussion

Plants have evolved a complicated and effective innate immune system to recognise, or respond to, many pathogenic organisms using *R* genes [1, 2]. At present, many *R* genes have been cloned from plants, and they can be divided into at least five classes based on conserved structural motifs, such as NBSs, LRRs and TIRs [4, 6]. In recent years, more than 20 plant genomes have been sequenced, and ~37,000 RGA genes were predicted based on conserved structural motifs [61]. Clearly, an analysis of the RGA genes in the genome will be useful for speculating on *R* gene evolution and for applying RGAs in cotton breeding. Recently, the genome of a diploid, *G. raimondii*, which is a Verticillium wilt-resistant wild relative of cotton, was sequenced [52, 53]. In this study, all probable RGA genes encoded by the *G. raimondii* genome were systematically analysed, and potential Verticillium wilt resistance loci/genes were identified using the bioinformatics analysis of transcriptome and QTL data.

In the *G. raimondii* genome, at least 300 genes encode NBS domains and most of these genes are of the CC-NBS or CC-NBS-LRR type [53, 56]. In this research, 1004 RGA genes were found in the *G. raimondii* genome based on an integrated annotation, and they were primarily distributed in Chr07, Chr09 and Chr11 (Additional file 2: Figure S1; Additional file 1: Table S1). As expected, the RGA genes showed a high similarity amongst themselves based on their conserved structural motifs, particularly when they occurred in small genomic regions of the same chromosome (Fig. 1, Additional file 1: Table S2). In contrast, some RGA genes in different families also showed similarities and were of the same phylogenetic lineage (Figs. 1 and 2). These results may indicate that the evolution of RGA genes in cotton had the dual characteristics of conservation and genetic variation, as did *RGC2* genes in lettuce [25]. RGA genes residing in clusters has been observed in many plant genomes [7, 10–14]. In *Arabidopsis thaliana*, more that 71 % of the NBS-LRR genes are arranged in 38 clusters [15], and the same characteristic is true of NBS-LRR genes in the rice genome [8]. As in other plants, the RGA genes in the *G. raimondii* genome reside in clusters (Fig. 3; Additional file 2: Figure S3; Additional file 1: Table S6). Previous studies have shown that the clustering of RGA genes is usually caused by tandem duplications [7, 62–64] or sequence exchanges [9], which have been detected in many RGA gene clusters [17, 19, 26, 65–67]. Similar results were found in the *G. raimondii* genome, where most of the RGA genes are homologous and linked together to form the Rgrcs (Additional file 2: Figure S2; Additional file 2: Figure S4; Additional file 1: Table S4), indicating that tandem duplication or sequence exchanges could have occurred frequently in the evolution of RGA genes or Rgrcs. Segmental duplication is another evolutionary mechanism in RGA genes that could randomly translocate the genes in chromosomes, giving rise to a substantial number of RGA genes [9, 28, 68]. This was also found in our analysis (Additional file 2: Figure S4B), probably suggesting that the segmental duplication could happen in the RGA genes evolution. Together, these results probably indicated that tandem duplication, sequence exchange, and segmental duplication are important to the evolution of RGA genes and Rgrcs.

Verticillium wilt is the most destructive disease in cotton, and there are no effective methods to prevent this disease at present. Although improving genetic resistance is the direct method to combat Verticillium wilt, it has not been successful in *G. hirsutum*, which accounts for more than 90 % of the total cotton acreage in the world, because of the lack of genetic resistance [38]. *G. barbadense* is considered to be a resistant species, and many studies regarding Verticillium wilt resistance have been reported [36, 43, 47–51]. Recently, a transcriptome analysis showed that some RGA genes were induced in *G. barbadense* inoculated with *V. dahliae* [42, 46], indicating that the RGA genes contribute to the defence response in *G. barbadense*. In this study, the RGA genes in the cotton response to *V. dahliae* were analysed using RNA-seq. To overcome problems caused by the complicated genome and high identities between RGAs, an extremely deep RNA-seq strategy was applied in this study to produce reliable DEG screening (Additional file 1: Table S7). The results showed that more DEGs were identified in this study compared with previous studies on *G. barbadense* infected with *V. dahliae* (Additional file 1: Table S8; Additional file 2: Figure S6) [42, 46], which suggests that deep sequencing is useful for the transcriptome analysis of cotton and particularly for the analysis of homologous genes. However, it must point out that the DEGs also possibility reflect diurnal or developmental regulation for various times inoculated samples compared with a single mock-inoculated sample in our experiment. qRT-PCR validation between the inoculated samples and their corresponding mock-inoculated controls is necessary for screening the Verticillium wilt response genes.

Plant genomes encode many RGA genes, and some of these genes are transcriptionally activated in the plant’s defence against pathogens [42, 46, 69–73]. Investigating the DEGs revealed that several hundred RGA genes, which belonged to different gene families, were induced in our experiment (Additional file 1: Table S10), and many of them were homologous to genes in the plant-pathogen interaction pathway (Fig. 5; Additional file 1: Table S11), which suggests that these RGA genes could participate in the defence response against Verticillium wilt. Moreover, the RGA genes strongly responded from 24 to 72 h (Additional file 2: Figure S8), which is an important infection stage in *V. dahliae* [57–59]. These results suggest that the expression of RGA genes is important to the defence response of Verticillium wilt resistance.

RGA genes that are distributed in gene clusters usually act as genetic resistance sources in plants [9, 74]. In the *G. raimondii* genome, the RGA genes in the Rgrcs were also induced, which most likely indicated that the RGA genes formed clusters that were involved in Verticillium wilt resistance (Fig. 6), similar to the resistance clusters in many other plants [75–78]. In this study, at least 26 potential VdRLs, which included 63 RGA genes, were found to be strongly induced in *G. barbadense*, and half of these loci were on Chr07 and Chr09 (Fig. 6; Additional file 1: Table S12-S14), which is consistent with a previous finding that VdRLs were mainly distributed on Chr07 and Chr09 in upland cotton [36]. Among these VdRLs, half were enriched for NB-ARC domain-encoding RGAs (Additional file 1: Table S15), which are involved in a variety of processes, including apoptosis, transcriptional regulation and effector-triggered immunity [79, 80]. Moreover, some RGAs that clustered in several VdRLs are homologous to pattern recognition receptors (Fig. 5; Additional file 1: Table S15), which suggests that the VdRLs, like cysteine-rich RLKs and receptor-like proteins, participate in PAMP-triggered immunity [2, 81, 82]. These results suggested that the mechanisms of cotton resistance to *V. dahliae* are complicated and require the participation of multiple RGAs or loci for cotton Verticillium wilt resistance.

To date, at least 80 different Verticillium wilt resistance QTLs have been reported in cotton [33–37]. With the bioinformatics analysis of the RGA’s distribution and expression after *V. dahliae* inoculation, at least 26 VdRLs were regarded as potential Verticillium wilt-response loci (Fig. 6). Interestingly, a correlation analysis showed that 12 VdRLs were less than 3 Mb (6 VdRLs were less than 1 Mb) from the closest Verticillium wilt resistance QTL, and 5 were of the NB-ARC gene cluster type (Fig. 7; Additional file 1: Table S14). An association analysis between disease resistance QTLs and NBS genes found that at least 32 NBS-encoding genes were adjacent to disease resistance QTLs in cotton [56], and there were similar results in other crops [56, 83–85]. Six of the VdRLs adjacent to Verticillium wilt resistance QTLs were located on the short region of Chr07 (Fig. 7; Additional file 1: Table S14), which again indicated that Verticillium wilt resistance QTLs clustered on chromosome D7 in cotton [36]. These results will be beneficial for understanding the VdRLs in cotton and cloning the Verticillium wilt resistance gene.

## Conclusions

In this study, the characteristics of RGA genes encoded in the *G. raimondii* genome were analysed, including the sequence structure, gene distribution and evolution. The *G. raimondii* genome encodes 1004 RGA genes, of which most are highly similar and could be clustered in HGs. Nearly half of the RGA genes occurred in 26 Rgrcs. Interestingly, many RGA genes are homologous, which results in most Rgrc sequences having a high similarity, indicating that sequence exchanges and tandem duplications frequently occurred in the evolution of RGA genes or Rgrcs. Moreover, the similarity among different Rgrcs suggests that some clusters may have evolved by segmental duplication. The RNA-seq analysis of the resistant cultivar *G. barbadense* showed that approximately half of the RGA genes were significantly induced by *V. dahliae* infection, and the portion of the RGA genes that formed 26 VdRLs in the Rgrcs were most likely involved in the Verticillium wilt response. A correlation analysis found that 12 VdRLs were adjacent to Verticillium wilt resistance QTLs, which strongly suggested that these loci respond during Verticillium wilt resistance in cotton.

## Methods

### Bioinformatics of RGA genes

Based on the integrated annotation of the *G. raimondii* reference genome from the DOE Joint Genome Institute (Cotton D V2.0, ftp://ftp.jgi-psf.org/pub/compgen/phytozome/v9.0/Graimondii/) [53], there were 11 classified RGA gene families, CC-NBS-LRR, cysteine-rich RLK, disease-resistance family protein/LRR family protein, leucine-rich receptor-like protein kinase family protein, LRR protein kinase family protein, LRR receptor-like protein kinase family protein, LRR transmembrane protein kinase, LRR and NB-ARC domain-containing disease resistance protein, NB-ARC domain-containing disease resistance protein, RLP and TIR-NBS-LRR.

The distribution of RGA genes in the *G. raimondii* genome was characterized by the number of RGA genes in the moving window (50 genes/window, walking forward 10 genes each time). The widows with RGA gene ratios that were greater than 10 % (considered Rgrcs) were collected and clusters only containing 6 RGA genes but distributed evenly were removed. Finally, the length of the Rgrcs was manually calculated based on the distribution of the RGA genes.

BLASTN and BLASTP programs (Version 2.2.23) were used to analyse the identities of the RGA genes (e ≤ 1e-10), using the best hit results for each RGA gene. The filtered results were used to construct an RGA gene matrix (total RGA genes versus total RGA genes) with a Perl script.

For the similarity analysis of RGA genes, a chimeric sequence was constructed by connecting RGA gene sequences in a series from Chr01 to Chr13. The similarities between segments of the chimeric sequences were analysed using the BLASTN program (Version 2.2.23), then small similarity blocks (less than the length of the smallest RGA gene, 216 bp) and the self-matching similarity blocks were removed. The homology between segments of the chimeric sequence was displayed using the ACT software [86]. The homology analysis of Rgrcs was performed using the same method, except similarity blocks less than 3 kb in length were filtered out.

In homology clustering, the reciprocal blast analysis of the proteins encoded by RGA genes (or encoding gene in Rgrcs) were conducted using the BLASTP program (Version 2.2.23) (e ≤ 1e-7). The clustering of gene families was performed as previously described [87] and the software Solar (Version 0.9.6) was used to remove redundant members (match rate < 33 % or identities < 30 %). Three other rigorous conditions (match rate < 70 % and identities < 70 %, match rate < 80 % and identities < 80 %, and match rate < 90 % and identities < 90 %) were also used for high homology analyses. The software hcluster_sg (Version 0.5.0) was used for gene family clustering. The homologous relationships among genes in Rgrcs were depicted using the Circos program (Version 0.64) [88].

To construct the phylogenetic tree of RGA genes, the MUSCLE program (Version 3.8.31) was applied to create multiple alignments of protein sequences [89]. The unrooted tree was generated using the TreeBeST program (Version 1.9.2) by the neighbour-joining method, with 1000 bootstrap replicates [90].

### Plant material and *V. dahliae* infection procedures

The resistant cultivar 7124 of *G. barbadense* L. was used as the experimental material. Cotton seeds were sown on commercial sterilised soil at 28 °C with a photoperiod of 14 h light/10 h dark for two weeks. Inoculations were performed using the high virulence V991 defoliating strain of *V. dahliae*. The strain was cultured on a potato-dextrose agar plate at 25 °C for one week. Spores were harvested from plates by eluting with sterile distilled water, then filtering through four layers of gauze and adjusted to 5 × 10^6^ spores/ml with sterile distilled water. The cotton two-week-old seedlings were inoculated with *V. dahliae* using the root dip method. Seedlings were gently uprooted, rinsed in sterile water, inoculated into a spore suspension for 10 min, and then returned to new pots containing sterilised soil. Six individual seedling roots were collected at six time points, 2, 6, 12, 24, 48 and 72 h after inoculation. Control plants were treated with sterile distilled water in the same way, and roots samples were immediately collected. All samples were immediately thrown into liquid nitrogen and stored at −80 °C until further analysis.

### Illumina sequencing

Total RNA was isolated from the root samples using an RNA kit according to the manufacturer’s instructions (EASYspin for plant RNA, Beijing, China). The seven RNA samples, including the samples from the six inoculation time points and the mock-inoculated, were used for RNA-seq. RNA samples were digested with DNase I (Qiagen, Hilden, Germany), and the quality and quantity were determined using a NanoDrop 2000 (Thermo Scientific, NH, USA) and an Agilent 2100 (Agilent, Santa Clara, CA, USA) instrument. RNA of each sample was purified using oligo(dT)-attached magnetic beads from an mRNA-Seq Sample Prep Kit (Illumina, San Diego, CA, USA). The purified mRNA was used for preparing a non-directional Illumina RNA-seq library using a Small RNA Sample Prep Kit (Illumina, San Diego, CA, USA). The library’s quality and quantification were analysed using an Agilent 2100 Bioanalyzer (Agilent, Santa Clara, CA, USA) and an ABI Step One Plus Real-Time PCR System (ABI, CA, USA). Each library was applied to an Illumina HiSeq 2000 (Illumina, San Diego, CA, USA) for single-end sequencing by the Beijing Genomics Institute (Shenzhen, China). Raw sequences were transformed into clean reads after data processing, leaving 49 nt tags.

### Mapping of Illumina reads against the *G. ramondii* genome

The raw FASTQ format data sets were produced from the software CASAVA v1.8.2, with quality controls. Reads contaminated with Illumina adapters were detected and removed, and high-quality reads (Phred score ≥ 20) were collected for further analysis. The software SOAPaligner/SOAP2.0 [91] was used to map reads to the reference sequence of the *G. ramondii* genome (DOE Joint Genome Institute: Cotton D V2.0, ftp://ftp.jgi-psf.org/pub/compgen/phytozome/v9.0/Graimondii/) [53], with less than two mismatches allowed in the alignment.

### Analysis of DEGs

The unique mapping read counts were normalised to RPKM, and the gene expression level was calculated using the RPKM method [92]. A strict algorithm was used to identify significant DEGs between mock-inoculated samples and inoculated samples. The FDR was set as 0.001 to determine the threshold of *p-*value (<0.001) in multiple tests, and the absolute value of log_2_Ratio was 1.0 [93]. The expression patterns were clustered using Cluster software [94]. The pathways were annotated based on the KEGG database [95] using BLASTX (e ≤ 1e-5). KEGG mapper and iPath tools were used for the plant-pathogen interaction pathway and the phytoalexin biosynthesis pathway analyses, respectively [95, 96].

### Quantitative RT-PCR analysis

A qRT-PCR analysis was performed using a two-step Real-Time PCR system (ABI Biosystems, CA, USA). New treatment samples were collected at six time points of 2, 6, 12, 24, 48 and 72 h after inoculation and their corresponding mock-inoculated controls. First-strand cDNA synthesis was performed with 2.0 μg of purified total RNA using the Superscript Reverse Transcriptase (Invitrogen, CA, USA). Gene-specific primers for qRT-PCR were designed using the Primer3 software (http://frodo.wi.mit.edu/primer3/) (Additional file 1: Table S17). The constitutively expressed cotton 18S gene was used for normalisation. The expression levels of 15 RGA genes were analysed using qRT-PCR with a SYBR Green PCR Master Mix according to the manufacturer’s instructions on an ABI 7500 Real Time PCR system (Applied Biosystems, CA, USA). The standard PCR cycles were as follows: 40 cycles at 95 °C for 30 s, 60 °C for 30 s, and 72 °C for 15 s. Three technical replicates for each sample were performed, and the relative quantification of gene expression levels was determined using the comparative Ct method [97].

### The correlation analysis between disease resistance QTL and VdRLs

The cotton Verticillium wilt resistance QTLs were retrieved from previous studies [33–37]. The primers and sequences of markers corresponding to these disease resistance QTLs were obtained from the Cotton Marker Database (http://www.cottonmarker.org). The physical locations of these QTLs in the diploid genome were determined by sequence mapping using PCR [98]. The physical distances between Verticillium wilt resistance QTLs and VdRLs in this study were calculated using their positions in the diploid cotton genome sequence mapping.

### Availability of supporting data

All relevant supporting data can be found within the supplementary files accompanying to this article. The Raw RNA-seq data supporting the results of this article is available through the Sequence Read Archive under accession NO. SRP03537 at website: http://www.ncbi.nlm.nih.gov/sra/?term=SRP035371. Phylogenetic data supporting the results of this article are available in the TreeBASE repository, http://purl.org/phylo/treebase/phylows/study/TB2:S17448.

## Additional files

Additional file 1: Table S1. Statistics of RGA genes in the *G. raimondii* genome. R-I–R-XI represents the 11 RGA gene families. **Table S2.** Analysis of the identities of RGA genes in Chr07 and Chr09. **Table S3.** Homology clustering of RGA genes in the *G. raimondii* genome. **Table S4.** Information regarding highly homologous genes screened by homology clustering. **Table S5.** Information on Rgrcs in the *G. raimondii* genome. **Table S6.** Statistical analysis of RGA genes in the Rgrcs. **Table S7.** Summary of sequencing yields and alignments. **Table S8.** Statistical analysis of DEGs in the *G. raimondii* genome and its RGA gene set. **Table S10.** Statistical analysis of DEGs in the 11 RGA gene families. **Table S11.** Information regarding differentially expressed RGA genes involved in the plant-pathogen interaction pathway. **Table S12.** Statistical analysis of potential DEGs in *G. barbadense* in response to *V. dahliae.* **Table S13.** Potential DEGs and VdRLs in *G. barbadense* in response to *V. dahliae*. **Table S14.** Information regarding VdRLs. **Table S15.** The RGA genes family enrichment in VdRLs. **Table S16.** Verticillium wilt resistance QTL information of cotton. **Table S17.** Primers used in this study. Additional file 2:
**Supplementary figures.** **Figure S1.** The statistics of RGA genes in *G. raimondii* chromosomes. The 11 families (R-I–R-XI) of RGA genes are cmarked in different colours. The X-axis represents the chromosomes of the *G. raimondii* genome (Chr01 to Chr13), and ‘Others’ represents the RGA genes that cannot be mapped to chromosomes at present. The Y-axis represents the number of genes. **Figure S2.** Homology clustering of RGA genes in the *G. raimondii* genome. Homology clustering was filtered using four conditions based on the match rates and identities among the RGA genes. Homology groups from HG01 to HG45 are arranged clockwise, the homology group intervals are differentiated by green and blue in series, according to the clustering conditions of match rate ≥ 33 % and identities ≥ 30 %. **Figure S3.** A sketch map of coding genes in Rgrcs. The coding genes in the Rgrcs are marked with a red line based on the physical map of the *G. raimondii* genome. For the genetic structures of the Rgrcs, RGA genes are represented by red squares and other genes are represented by black squares. **Figure S4.** Homology analysis of Rgrcs in the *G. raimondii* genome. (A) Homology analysis of the Rgrcs’ chimeric sequence. The chimeric sequence connected the Rgrc sequences in a series from Chr01 to Chr13 and was compared using the BlastN program (Version 2.2.23), ignoring self-matches and filtering out similarity blocks less than 3 kb in length. The forward-forward matches are marked with red lines, and the forward-reverse matches are marked with blue lines. (B) A statistical analysis of the similarity blocks among 26 Rgrcs. The lengths of the similarity blocks is greater than 3 kb. (C) Distribution of the identities of homology blocks. **Figure S5.** Cotton inoculated with *V. dahliae*. Two-week-old seedlings of the resistant cultivar *G. barbadense* cv. 7124 and the susceptible cultivar *G. hirsutum* cv. Jummian1 inoculated with the high virulence V991 defoliating strain of *V. dahliae* (5 × 10^6^ spores/ml). The phenotypes were investigated three weeks after inoculation in this study. **Figure S6.** Statistical analysis of the DEGs in cotton inoculated with *V. dahliae*. The left side is the DEGs of all the genes in the *G. raimondii* genome and the right side is the RGA gene set. The X-axis represents numbers of DEGs. ‘I2–I72’ represents the six inoculation time points (in hours). The numbers in the brackets from left to right represent the number of DEGs with more than a two-fold and a four-fold change, respectively, compared with mock-inoculated,. Red represents up-regulation and green represents down-regulation. **Figure S7.** Differentially expressed RGA genes confirmed by qRT-PCR. In total, 15 differentially expressed RGA genes were randomly selected for qRT-PCR validation. The left side is the DEGs determined using RNA-seq and right side is the validation results using qRT-PCR. **Figure S8.** Expression pattern analysis of the response of 11 RGA gene families to *V. dahliae*. The filter conditions are FDR < 0.001 and *p* < 0.001. R-I–R-XI represents the 11 RGA gene families. ‘I2–I72’ represents the six inoculation time points. **Figure S9.** Phytoalexin biosynthesis pathway of *G. barbadense* inoculated with *V. dahliae*. The DEGs used for the metabolism pathway analysis were screened by FDR < 0.001, *p* < 0.001, and log_2_Ratio ≥ |1.0| at all six inoculation time points. The thin lines represent the expression change of log_2_Ratio ≥ |1.0|, and the thick lines represent the expression change of log_2_Ratio ≥ |2.0|. **Figure S10.** Clustering of DEGs encoded in Rgrcs. (A) The expression pattern analysis of RGA genes in Rgrcs. (B) The expression pattern analysis of other genes, not encoding RGA genes in Rgrcs. Additional file 3: Table S9. DEGs of *G. barbadense* inoculated with *V. dahliae*.

## Abbreviations

RGA: Resistance Gene Analogue
Rgrc: RGA-gene-rich cluster
VdRL: *V. dahliae* response loci
HG: Homology groups
DEG: Differentially expression gene
QTL: Quantitative trait locus
