# Supplementary material for: Genome-wide analysis of the gene families of resistance gene analogues in cotton and their response to Verticillium wilt
Source: BMC Plant Biol. 2015 Jun 19;15:148. doi: 10.1186/s12870-015-0508-3 (PMC4471920; doi:10.1186/s12870-015-0508-3)
Supplement: Additional file 1: Table S1. — Statistics of RGA genes in the G. raimondii genome. R-I–R-XI represents the 11 RGA gene families. Table S2. Analysis of the identities of RGA genes in Chr07 and Chr09. Table S3. Homology clustering of RGA genes in the G. raimondii genome. Table S4. Information regarding highly homologous genes screened by homology clustering. Table S5. Information on Rgrcs in the G. raimondii genome. Table S6. Statistical analysis of RGA genes in the Rgrcs. Table S7. Summary of sequencing yields and alignments. Table S8. Statistical analysis of DEGs in the G. raimondii genome and its RGA gene set. Table S10. Statistical analysis of DEGs in the 11 RGA gene families. Table S11. Information regarding differentially expressed RGA genes involved in the plant-pathogen interaction pathway. Table S12. Statistical analysis of potential DEGs in G. barbadense in response to V. dahliae. Table S13. Potential DEGs and VdRLs in G. barbadense in response to V. dahliae. Table S14. Information regarding VdRLs. Table S15. The RGA genes family enrichment in VdRLs. Table S16. Verticillium wilt resistance QTL information of cotton. Table S17. Primers used in this study. [file 12870_2015_508_MOESM1_ESM.pdf]

Table S1 Statistics of RGA genes in the *G. raimondii* genome

Family ID	RGA gene type#	Total No.
R-I	CC-NBS-LRR	32
R-II	Cysteine-rich RLK	60
R-III	Disease resistance family protein / LRR family protein	46
R-IV	Leucine-rich receptor-like protein kinase family protein	58
R-V	Leucine-rich repeat protein kinase family protein	225
R-VI	Leucine-rich repeat receptor-like protein kinase family protein	44
R-VII	Leucine-rich repeat transmembrane protein kinase	78
R-VIII	LRR and NB-ARC domains-containing disease resistance protein	79
R-IX	NB-ARC domain-containing disease resistance protein	194
R-X	Receptor like protein	144
R-XI	TIR-NBS-LRR	44

#note: the classify of RGA gene types based on the integrated annotation of *G. ramondii* genome [53]

Table S2 Analysis of the identities of RGA genes in Chr07 and Chr09

	Gorai.007G335800.1	Gorai.007G336000.1	Gorai.007G336100.1	Gorai.007G336200.1	Gorai.007G337100.1	Gorai.007G337200.1	Gorai.007G337300.1	Gorai.007G337400.1	Gorai.007G337500.1	Gorai.007G338100.1	Gorai.007G338300.1	Gorai.007G338400.1	Gorai.007G338700.1	Gorai.007G338800.1	Gorai.007G338900.1	Gorai.007G339000.1	Gorai.007G339100.1	Gorai.007G339200.1	Gorai.007G339300.1	Gorai.007G339400.1	Gorai.007G339600.1	Gorai.007G339700.1	Gorai.007G339800.1	Gorai.007G339900.1	Gorai.007G340000.1	Gorai.007G340100.1	Gorai.007G340200.1	Gorai.007G340300.1	Gorai.007G340700.1
Gorai.007G335800.1	100	94	94	93	92	92	95	95	90	93	87	92	95	94	84	93	88	88	84	85	85	89	89	86	87	82	85	85	84
Gorai.007G336000.1	94	100	91	91	91	90	95	94	89	93	86	90	95	93	83	95	88	89	83	92	85	89	92	83	87	81	84	92	83
Gorai.007G336100.1	94	91	100	95	93	94	94	94	93	93	90	94	96	97	92	92	90	83	85	85	89	91	90	85	87	84	87	92	90
Gorai.007G336200.1	93	91	95	100	93	95	94	93	91	93	89	93	94	92	88	94	90	93	84	85	90	90	0	85	87	86	87	91	84
Gorai.007G337100.1	92	91	93	93	100	96	95	92	93	94	95	93	97	98	92	96	90	97	88	91	89	91	87	89	91	89	91	91	87
Gorai.007G337200.1	92	90	94	95	96	100	95	93	95	94	94	92	94	91	91	94	91	96	86	85	88	90	85	87	88	88	90	90	87
Gorai.007G337300.1	95	95	97	94	95	95	100	92	93	95	87	95	95	94	83	93	88	88	82	87	85	92	88	83	86	82	85	82	82
Gorai.007G337400.1	95	94	94	93	92	93	92	100	91	91	90	92	93	92	91	97	92	91	84	85	90	90	85	86	88	92	88	95	90
Gorai.007G337500.1	90	89	93	91	93	95	93	91	100	94	88	92	91	89	93	93	90	0	87	86	89	90	0	88	90	87	91	90	88
Gorai.007G338100.1	93	93	93	93	94	94	95	91	94	100	88	92	94	92	83	95	88	89	89	91	87	91	87	90	87	82	85	93	83
Gorai.007G338300.1	87	86	90	89	95	94	87	90	88	90	100	89	95	94	90	93	88	88	87	90	91	90	91	89	90	88	90	87	86
Gorai.007G338400.1	92	90	94	93	93	92	89	92	92	92	89	100	93	95	92	92	94	86	88	89	91	91	87	89	92	88	87	90	89
Gorai.007G338700.1	95	96	96	94	97	94	95	93	91	94	94	93	100	96	92	95	88	89	85	86	87	90	88	86	88	89	86	91	84
Gorai.007G338800.1	94	93	97	92	95	91	94	92	89	92	94	94	96	100	92	94	90	88	87	96	91	88	88	88	92	89	92	91	86
Gorai.007G338900.1	84	83	92	88	92	91	83	91	93	83	90	92	92	92	100	91	86	88	84	88	84	88	89	85	91	90	84	87	84
Gorai.007G339000.1	93	95	92	94	96	94	93	97	93	95	92	92	95	94	91	100	88	96	87	92	89	86	87	88	90	88	90	90	88
Gorai.007G339100.1	88	88	90	90	90	91	88	92	90	88	88	94	88	90	86	88	100	92	84	87	86	87	88	86	92	91	83	84	85
Gorai.007G339200.1	88	89	81	93	97	96	88	91	0	89	88	86	89	88	88	96	92	100	87	0	90	91	90	87	94	86	87	89	87
Gorai.007G339300.1	84	83	85	84	88	86	82	84	87	89	87	88	85	87	84	87	84	87	100	83	83	84	89	92	84	90	82	82	95
Gorai.007G339400.1	85	92	85	85	91	85	87	85	86	91	90	89	86	96	88	92	87	0	83	100	91	86	0	86	85	85	87	88	82
Gorai.007G339600.1	85	85	89	90	89	90	85	90	89	87	91	91	86	91	84	91	86	90	83	91	100	87	88	88	89	85	89	87	83
Gorai.007G339700.1	89	89	91	90	91	90	92	90	90	91	90	91	90	88	88	86	87	91	84	86	87	100	86	86	92	86	87	85	84
Gorai.007G339800.1	89	92	90	0	87	85	88	85	0	87	91	87	88	88	89	87	88	90	89	0	88	86	100	88	89	88	88	87	88
Gorai.007G339900.1	86	83	85	85	89	87	83	86	88	90	89	89	86	88	85	88	86	87	92	86	88	86	88	100	90	96	82	87	92
Gorai.007G340000.1	87	87	87	87	91	88	86	88	90	90	90	92	88	92	91	91	92	94	84	85	89	92	89	90	100	88	89	87	88
Gorai.007G340100.1	82	81	84	86	89	88	82	92	87	82	88	88	89	89	90	88	91	86	90	85	85	86	88	96	88	100	88	87	90
Gorai.007G340200.1	85	84	87	87	91	92	85	88	91	88	90	87	92	92	84	92	83	87	82	87	89	87	88	82	89	88	100	87	82
Gorai.007G340300.1	85	92	92	91	91	90	82	95	90	93	87	90	91	91	87	90	84	89	82	88	87	85	87	87	87	87	87	100	82
Gorai.007G340700.1	84	83	90	84	87	87	82	90	88	83	86	89	84	86	84	88	85	87	95	82	83	84	88	92	88	90	82	82	100

	Gorai.009G375100.1	Gorai.009G379400.1	Gorai.009G379500.1	Gorai.009G379600.1	Gorai.009G379700.1	Gorai.009G379800.1	Gorai.009G379900.1	Gorai.009G380000.1	Gorai.009G380100.1	Gorai.009G380200.1	Gorai.009G380300.1	Gorai.009G380400.1	Gorai.009G380500.1	Gorai.009G380600.1	Gorai.009G380700.1	Gorai.009G381300.1	Gorai.009G381500.1	Gorai.009G381800.1	Gorai.009G382200.1	Gorai.009G382700.1	Gorai.009G383000.1	Gorai.009G383200.1	Gorai.009G385200.1	Gorai.009G385400.1	Gorai.009G385500.1	Gorai.009G385600.1	Gorai.009G385800.1	Gorai.009G386200.1	Gorai.009G386400.1	Gorai.009G386500.1	Gorai.009G386600.1	Gorai.009G386700.1	Gorai.009G387300.1	Gorai.009G387500.1	Gorai.009G387600.1
Gorai.009G375100.1	100	80	80	84	83	85	83	85	86	84	82	85	84	85	86	84	84	85	85	84	84	83	84	81	83	83	81	84	84	84	85	84	87	85	84
Gorai.009G379400.1	80	100	83	92	89	91	92	89	90	92	88	0	91	0	91	93	90	91	95	90	92	88	88	87	0	90	91	92	89	90	0	92	91	91	91
Gorai.009G379500.1	80	83	100	86	86	85	87	83	84	86	86	0	83	0	88	86	84	85	82	83	84	85	84	86	0	84	85	87	88	86	0	85	83	84	84
Gorai.009G379600.1	84	92	86	100	91	89	92	92	89	92	93	95	92	93	95	93	95	89	90	95	96	94	93	90	94	93	92	90	90	89	92	96	92	89	93
Gorai.009G379700.1	83	89	86	91	100	89	94	90	89	93	92	91	89	91	91	91	89	89	92	91	90	90	90	89	88	92	90	91	90	89	98	90	88	89	91
Gorai.009G379800.1	85	91	85	89	89	100	89	90	96	90	92	90	89	92	90	90	89	94	89	89	91	88	88	89	93	90	90	91	90	90	90	90	96	94	91
Gorai.009G379900.1	83	92	87	92	94	89	100	91	89	94	89	93	90	92	92	92	90	91	92	90	92	90	89	89	91	92	90	90	91	92	95	91	89	91	92
Gorai.009G380000.1	85	89	83	92	90	90	91	100	90	93	92	94	91	95	89	91	91	90	90	91	93	90	90	91	95	90	96	91	90	89	92	91	92	90	93
Gorai.009G380100.1	86	90	84	89	89	96	89	90	100	89	93	90	87	91	91	89	88	96	91	90	91	86	88	90	92	89	91	90	90	91	90	90	97	96	91
Gorai.009G380200.1	84	92	86	92	93	90	94	93	89	100	91	94	92	97	95	91	91	90	92	92	93	91	90	90	93	92	94	92	94	91	92	92	92	89	92
Gorai.009G380300.1	82	88	86	93	92	92	89	92	93	91	100	0	96	0	91	89	90	92	92	89	89	94	91	92	0	90	91	96	91	92	0	92	92	92	92
Gorai.009G380400.1	85	0	0	95	91	90	93	94	90	94	0	100	93	94	95	95	95	90	93	94	95	96	96	0	94	94	95	91	91	93	92	96	93	90	94
Gorai.009G380500.1	84	91	83	92	89	89	90	91	87	92	96	93	100	95	98	90	90	92	89	90	92	90	93	91	95	89	92	88	90	92	91	91	89	93	92
Gorai.009G380600.1	85	0	0	93	91	92	92	95	91	97	0	94	95	100	95	96	94	93	93	97	96	93	94	0	93	90	95	93	95	90	91	94	92	91	95
Gorai.009G380700.1	86	91	88	95	91	90	92	89	91	95	91	95	98	95	100	93	89	90	90	89	90	91	89	90	96	89	90	90	90	93	92	90	90	91	96
Gorai.009G381300.1	84	93	86	93	91	90	92	91	89	91	89	95	90	96	93	100	93	90	89	89	91	96	89	90	0	89	90	90	89	91	88	91	89	90	96
Gorai.009G381500.1	84	90	84	95	89	89	90	91	88	91	90	95	90	94	89	93	100	89	89	91	92	95	92	88	94	90	91	90	89	88	91	92	91	90	93
Gorai.009G381800.1	85	91	85	89	89	94	91	90	96	90	92	90	92	93	90	90	89	100	90	90	91	87	90	90	94	90	91	91	90	90	89	91	91	94	91
Gorai.009G382200.1	85	95	82	90	92	89	92	90	91	92	92	93	89	93	90	89	89	90	100	89	90	88	88	88	0	90	91	91	91	91	99	90	91	91	90
Gorai.009G382700.1	84	90	83	95	91	89	90	91	90	92	89	94	90	97	89	89	91	90	89	100	93	94	90	88	94	91	93	91	90	89	92	93	89	90	94
Gorai.009G383000.1	84	92	84	96	90	91	92	93	91	93	89	95	92	96	90	91	92	91	90	93	100	95	91	89	94	91	92	92	90	90	92	94	89	91	95
Gorai.009G383200.1	83	88	85	94	90	88	90	90	86	91	94	96	90	93	91	96	95	87	88	94	95	100	90	91	91	92	91	88	89	91	91	93	90	88	92
Gorai.009G385200.1	84	88	84	93	90	88	89	90	88	90	91	96	93	94	89	89	92	90	88	90	91	90	100	90	95	89	91	88	88	90	92	91	90	91	92
Gorai.009G385400.1	81	87	86	90	89	89	89	91	90	90	92	0	91	0	90	90	88	90	88	88	89	91	90	100	0	90	90	90	90	91	0	88	89	93	89
Gorai.009G385500.1	83	0	0	94	88	93	91	95	92	93	0	94	95	93	96	0	94	94	0	94	94	91	95	0	100	91	94	90	0	93	89	92	92	92	95
Gorai.009G385600.1	83	90	84	93	92	90	92	90	89	92	90	94	89	90	89	89	90	90	90	91	91	92	89	90	91	100	92	91	91	89	96	92	91	90	92
Gorai.009G385800.1	81	91	85	92	90	90	90	96	91	94	91	95	92	95	90	90	91	91	91	93	92	91	91	90	94	92	100	92	92	90	93	93	91	91	97
Gorai.009G386200.1	84	92	87	90	91	91	90	91	90	92	96	91	88	93	90	90	90	91	91	91	92	88	88	90	90	91	92	100	94	89	93	92	91	91	92
Gorai.009G386400.1	84	89	88	90	90	90	91	90	90	94	91	91	90	95	90	89	89	90	91	90	90	89	88	90	0	91	92	94	100	90	96	90	89	91	91
Gorai.009G386500.1	84	90	86	89	89	90	92	89	91	91	92	93	92	90	93	91	88	90	91	89	90	91	90	91	93	89	90	89	90	100	89	90	91	91	91
Gorai.009G386600.1	85	0	0	92	98	90	95	92	90	92	0	92	91	91	92	0	91	89	99	92	92	91	92	0	89	96	93	93	96	89	100	93	89	90	93
Gorai.009G386700.1	84	92	85	96	90	90	91	91	90	92	92	96	91	94	90	91	92	91	90	93	94	93	91	88	92	92	93	92	90	90	93	100	91	90	95
Gorai.009G387300.1	87	91	83	92	89	90	89	92	97	92	92	93	89	92	90	89	91	91	91	89	89	90	90	89	92	91	91	91	89	91	89	91	100	92	93
Gorai.009G387500.1	85	91	84	89	89	94	91	90	96	89	92	90	93	91	91	90	90	94	91	90	91	88	91	93	92	90	91	91	91	91	90	90	92	100	91
Gorai.009G387600.1	84	91	84	93	91	91	92	93	91	92	92	94	92	95	96	96	93	91	90	94	95	92	92	89	95	92	97	92	91	91	93	95	93	91	100

Table S3 Homology clustering of RGA genes in the *G. raimondii* genome

Family[#]	R-I	R-II	R-III	R-IV	R-V	R-VI	R-VII	R-VIII	R-IX	R-X	R-XI	Total
HG01	0	0	0	0	0	0	0	0	0	0	30	30
HG02	0	0	0	0	0	0	0	0	0	0	4	4
HG03	0	0	0	0	0	0	0	32	54	0	0	86
HG04	0	0	0	0	0	0	0	1	1	0	0	2
HG05	8	0	0	0	0	0	0	1	21	0	0	30
HG06	2	0	0	0	0	0	0	0	0	0	0	2
HG07	0	0	0	0	0	0	0	0	4	0	0	4
HG08	0	0	0	0	0	0	0	1	4	0	0	5
HG09	0	0	0	0	0	0	0	0	2	0	0	2
HG10	12	0	0	0	0	0	0	20	47	0	0	79
HG11	2	0	0	0	0	0	0	3	27	0	1	33
HG12	2	0	0	0	0	0	0	5	4	0	0	11
HG13	2	0	0	0	0	0	0	5	16	0	0	23
HG14	0	0	0	0	0	0	0	0	2	0	0	2
HG15	0	0	0	0	0	0	0	8	0	0	1	9
HG16	0	0	0	0	0	0	0	1	1	0	0	2
HG17	0	0	0	45	112	37	48	0	0	0	0	242
HG18	0	0	0	0	8	0	0	0	0	0	0	8
HG19	0	0	0	9	12	4	5	0	0	5	0	35
HG20	0	0	42	1	0	0	1	0	0	116	0	160
HG21	0	0	0	0	56	0	3	0	0	0	0	59
HG22	0	50	0	0	9	0	2	0	0	0	0	61
HG23	0	0	0	0	10	0	0	0	0	0	0	10
HG24	0	0	0	0	8	0	0	0	0	0	0	8
HG25	0	0	0	0	0	2	0	0	0	0	0	2
HG26	0	0	0	0	3	0	0	0	0	0	0	3
HG27	0	3	0	0	0	0	0	0	0	0	0	3
HG28	0	7	0	0	0	0	0	0	0	0	0	7
HG29	0	0	0	2	0	0	0	0	0	0	0	2
HG30	0	0	3	0	0	0	0	0	0	0	0	3
HG31	0	0	1	0	0	0	0	0	0	1	0	2
HG32	0	0	0	0	0	0	0	0	0	3	0	3
HG33	0	0	0	0	1	0	0	0	0	2	0	3
HG34	0	0	0	0	2	0	0	0	0	3	0	5
HG35	0	0	0	0	0	0	6	0	0	0	0	6
HG36	0	0	0	0	0	0	4	0	0	0	0	4
HG37	0	0	0	0	0	0	0	0	0	3	0	3
HG38	0	0	0	0	0	0	0	0	0	5	0	5
HG39	0	0	0	0	0	0	3	0	0	0	0	3
HG40	0	0	0	0	0	0	0	0	0	2	0	2
HG41	0	0	0	0	0	0	0	0	0	0	2	2
HG42	0	0	0	0	0	0	0	0	0	0	3	3
HG43	0	0	0	0	0	0	0	0	2	0	0	2
HG44	2	0	0	0	0	0	0	0	0	0	0	2
HG45	0	0	0	0	0	0	0	2	0	0	0	2
NA	2	0	0	1	4	1	6	0	9	4	3	30

[#] HG, homology group, clustered with Match rate≥33% and identities≥30%, green box represents the HG up to 20 genes

Table S4 Information regarding highly homologous genes screened by homology clustering

Sub-Homology groups	Gene ID	Functional annotation
HG01-01	Gorai.003G172400.1<br>Gorai.003G173100.1<br>Gorai.003G173200.1<br>Gorai.003G173400.1<br>Gorai.003G173800.1<br>Gorai.003G174500.1	Leucine-rich receptor-like protein kinase family protein
HG01-02	Gorai.003G172700.1<br>Gorai.003G174400.1	Leucine-rich receptor-like protein kinase family protein
HG01-03	Gorai.007G318500.1<br>Gorai.007G318900.1	Leucine-rich receptor-like protein kinase family protein
HG02-01	Gorai.007G318700.1<br>Gorai.007G319000.1	Leucine-rich receptor-like protein kinase family protein
HG05-01	Gorai.007G332600.1<br>Gorai.007G332900.1<br>Gorai.NG010200.1	Receptor like protein<br>Disease resistance family protein / LRR family protein
HG05-02	Gorai.007G333200.1<br>Gorai.007G333300.1<br>Gorai.007G333800.1<br>Gorai.007G333900.1<br>Gorai.007G334100.1<br>Gorai.NG010100.1	Receptor like protein
HG05-03	Gorai.011G270100.1<br>Gorai.011G270400.1	Disease resistance family protein / LRR family protein
HG05-04	Gorai.007G324100.1<br>Gorai.007G324200.1<br>Gorai.007G324300.1<br>Gorai.007G324400.1<br>Gorai.007G324600.1<br>Gorai.007G324700.1<br>Gorai.007G325100.1	Receptor like protein<br>Disease resistance family protein / LRR family protein
HG10-01	Gorai.009G441400.1<br>Gorai.009G441500.1	NB-ARC domain-containing disease resistance protein
HG10-02	Gorai.010G225200.1<br>Gorai.010G225700.1	NB-ARC domain-containing disease resistance protein
HG10-03	Gorai.002G018500.1<br>Gorai.002G018600.1<br>Gorai.002G018700.1<br>Gorai.002G018900.1<br>Gorai.002G019200.1<br>Gorai.002G019500.1	Disease resistance family protein / LRR family protein<br>NB-ARC domain-containing disease resistance protein
HG10-04	Gorai.002G044500.1<br>Gorai.002G044900.1	Disease resistance family protein / LRR family protein<br>Receptor like protein
	Gorai.003G073100.1<br>Gorai.009G379600.1<br>Gorai.009G379700.1<br>Gorai.009G379800.1<br>Gorai.009G379900.1<br>Gorai.009G380000.1<br>Gorai.009G380100.1<br>Gorai.009G380200.1	

Sub-Homology groups	Gene ID	Functional annotation
HG10-05	Gorai.009G380500.1<br>Gorai.009G381300.1<br>Gorai.009G381500.1<br>Gorai.009G381800.1<br>Gorai.009G382200.1<br>Gorai.009G382700.1<br>Gorai.009G383000.1<br>Gorai.009G383200.1<br>Gorai.009G385200.1<br>Gorai.009G385600.1<br>Gorai.009G385800.1<br>Gorai.009G386200.1<br>Gorai.009G386400.1<br>Gorai.009G386500.1<br>Gorai.009G386700.1<br>Gorai.009G387500.1<br>Gorai.009G387600.1<br>Gorai.009G430600.1	Receptor like protein
HG10-06	Gorai.009G380700.1<br>Gorai.009G387300.1	Receptor like protein
HG10-07	Gorai.007G320300.1<br>Gorai.007G320500.1	NB-ARC domain-containing disease resistance protein<br>Receptor like protein
HG11-01	Gorai.009G435900.1<br>Gorai.009G436500.1<br>Gorai.009G436900.1	Disease resistance family protein / LRR family protein<br>Receptor like protein
HG12-01	Gorai.009G380400.1<br>Gorai.009G380600.1	Disease resistance family protein / LRR family protein<br>NB-ARC domain-containing disease resistance protein
HG12-02	Gorai.009G386600.1<br>Gorai.009G434100.1	Receptor like protein<br>NB-ARC domain-containing disease resistance protein
HG13-01	Gorai.011G263000.1<br>Gorai.011G264800.1<br>Gorai.011G265900.1	Receptor like protein<br>NB-ARC domain-containing disease resistance protein
HG15-01	Gorai.011G263200.1<br>Gorai.011G264200.1	NB-ARC domain-containing disease resistance protein
HG15-02	Gorai.011G263700.1<br>Gorai.011G263900.1	NB-ARC domain-containing disease resistance protein
HG17-01	Gorai.011G284600.1<br>Gorai.011G285100.1<br>Gorai.011G284400.1	Leucine-rich repeat receptor-like protein kinase family protein
HG17-02	Gorai.011G258500.1<br>Gorai.011G259200.1<br>Gorai.011G260200.1<br>Gorai.011G260300.1<br>Gorai.011G258700.1<br>Gorai.011G259500.1<br>Gorai.011G259800.1	Leucine-rich repeat receptor-like protein kinase family protein
HG17-03	Gorai.013G036900.1<br>Gorai.008G029000.1	Leucine-rich repeat receptor-like protein kinase family protein
HG17-04	Gorai.010G040900.1<br>Gorai.010G041800.1	Leucine-rich repeat transmembrane protein kinase
HG17-05	Gorai.009G159400.1<br>Gorai.009G159500.1	LRR and NB-ARC domains-containing disease resistance protein

Sub-Homology groups	Gene ID	Functional annotation
HG17-06	Gorai.009G237700.1<br>Gorai.NG013500.1	Leucine-rich repeat transmembrane protein kinase
HG17-07	Gorai.009G306900.1<br>Gorai.009G307100.1	Leucine-rich repeat receptor-like protein kinase family protein
HG17-08	Gorai.009G365300.1<br>Gorai.009G370000.1<br>Gorai.009G370300.1	Leucine-rich repeat receptor-like protein kinase family protein
HG17-09	Gorai.009G435400.1<br>Gorai.009G436200.1<br>Gorai.009G435600.1<br>Gorai.009G436300.1	Leucine-rich repeat receptor-like protein kinase family protein
HG17-10	Gorai.010G218400.1<br>Gorai.011G209800.1	Leucine-rich repeat receptor-like protein kinase family protein
HG17-11	Gorai.011G217500.1<br>Gorai.011G217600.1	Leucine-rich repeat transmembrane protein kinase
HG17-12	Gorai.002G048300.1<br>Gorai.002G048600.1<br>Gorai.002G048800.1	LRR and NB-ARC domains-containing disease resistance protein
HG17-13	Gorai.002G049000.1<br>Gorai.009G363600.1	LRR and NB-ARC domains-containing disease resistance protein
HG17-14	Gorai.002G051100.1<br>Gorai.002G051900.1	Leucine-rich repeat receptor-like protein kinase family protein
HG17-15	Gorai.002G051300.1<br>Gorai.002G051700.1	Leucine-rich repeat receptor-like protein kinase family protein
HG17-16	Gorai.002G103300.1<br>Gorai.009G452000.1	Leucine-rich repeat protein kinase family protein
HG17-17	Gorai.002G131000.1<br>Gorai.002G133000.1	Leucine-rich repeat receptor-like protein kinase family protein
HG17-18	Gorai.002G149600.1<br>Gorai.003G048200.1<br>Gorai.007G062800.1	Leucine-rich repeat protein kinase family protein
HG17-19	Gorai.002G217000.1<br>Gorai.005G265400.1<br>Gorai.008G045400.1	Leucine-rich repeat protein kinase family protein
HG17-20	Gorai.003G034400.1<br>Gorai.003G034500.1<br>Gorai.007G228700.1<br>Gorai.008G087500.1	Leucine-rich repeat protein kinase family protein
HG17-21	Gorai.003G039900.1<br>Gorai.007G060500.1<br>Gorai.007G236500.1	LRR and NB-ARC domains-containing disease resistance protein
HG17-22	Gorai.003G046400.1<br>Gorai.007G244000.1	Leucine-rich repeat protein kinase family protein
HG17-23	Gorai.003G092400.1<br>Gorai.006G137100.1	Leucine-rich repeat receptor-like protein kinase family protein
HG17-24	Gorai.004G204100.1<br>Gorai.008G241300.1	Leucine-rich repeat receptor-like protein kinase family protein
HG17-25	Gorai.001G186800.1<br>Gorai.004G225900.1	Leucine-rich repeat receptor-like protein kinase family protein
HG17-26	Gorai.004G249900.1<br>Gorai.007G271300.1	Leucine-rich repeat receptor-like protein kinase family protein

Sub-Homology groups	Gene ID	Functional annotation
HG17-27	Gorai.010G096300.1<br>Gorai.001G019800.1	Leucine-rich repeat protein kinase family protein
HG17-28	Gorai.001G049500.1<br>Gorai.008G215400.1	Leucine-rich repeat receptor-like protein kinase family protein
HG17-29	Gorai.001G074000.1<br>Gorai.007G035200.1	Leucine-rich repeat receptor-like protein kinase family protein
HG17-30	Gorai.011G052300.1<br>Gorai.011G052600.1<br>Gorai.001G131900.1	LRR and NB-ARC domains-containing disease resistance protein
HG17-31	Gorai.011G052400.1<br>Gorai.NG011200.1	LRR and NB-ARC domains-containing disease resistance protein
HG17-32	Gorai.011G052000.1<br>Gorai.011G052100.1	LRR and NB-ARC domains-containing disease resistance protein
HG17-33	Gorai.001G181900.1<br>Gorai.001G237400.1	LRR and NB-ARC domains-containing disease resistance protein
HG17-34	Gorai.005G039600.1<br>Gorai.006G219400.1	Leucine-rich repeat transmembrane protein kinase
HG17-35	Gorai.012G097800.1<br>Gorai.009G008400.1	Leucine-rich repeat receptor-like protein kinase family protein
HG17-36	Gorai.006G019800.1<br>Gorai.006G020000.1	Leucine-rich repeat transmembrane protein kinase
HG17-37	Gorai.006G020100.1<br>Gorai.006G020400.1	Leucine-rich repeat transmembrane protein kinase
HG17-38	Gorai.007G330300.1<br>Gorai.007G330600.1<br>Gorai.007G330700.1	LRR and NB-ARC domains-containing disease resistance protein
HG19-01	Gorai.009G365500.1<br>Gorai.009G366100.1<br>Gorai.009G371700.1	LRR and NB-ARC domains-containing disease resistance protein<br>Leucine-rich repeat protein kinase family protein
HG19-02	Gorai.009G370200.1<br>Gorai.NG027000.1	Leucine-rich repeat receptor-like protein kinase family protein
HG19-03	Gorai.010G041100.1<br>Gorai.002G247500.1	Leucine-rich repeat transmembrane protein kinase
HG19-04	Gorai.007G328700.1<br>Gorai.007G329000.1<br>Gorai.007G329200.1	Leucine-rich repeat receptor-like protein kinase family protein<br>Leucine-rich repeat protein kinase family protein
HG20-01	Gorai.007G335800.1<br>Gorai.007G336000.1<br>Gorai.007G337200.1<br>Gorai.007G338100.1<br>Gorai.007G338700.1	Cysteine-rich RLK
HG20-02	Gorai.007G339300.1<br>Gorai.007G339900.1<br>Gorai.007G340700.1	Cysteine-rich RLK
HG20-03	Gorai.009G367500.1<br>Gorai.009G367700.1<br>Gorai.009G367800.1	TIR-NBS-LRR
HG20-04	Gorai.009G444400.1<br>Gorai.009G444500.1<br>Gorai.009G447900.1<br>Gorai.009G448000.1<br>Gorai.009G448100.1	TIR-NBS-LRR

Sub-Homology groups	Gene ID	Functional annotation
	Gorai.009G448200.1<br>Gorai.009G448600.1	
HG20-05	Gorai.011G143400.1<br>Gorai.011G143500.1	Cysteine-rich RLK
HG20-06	Gorai.010G203200.1<br>Gorai.004G023800.1	TIR-NBS-LRR
HG20-07	Gorai.005G026300.1<br>Gorai.005G026600.1<br>Gorai.005G029300.1	TIR-NBS-LRR
HG20-08	Gorai.012G117000.1<br>Gorai.012G117200.1	Receptor like protein
HG20-09	Gorai.007G324500.1<br>Gorai.007G324800.1	TIR-NBS-LRR
HG20-10	Gorai.007G327300.1<br>Gorai.007G327600.1<br>Gorai.007G327800.1<br>Gorai.007G328000.1<br>Gorai.007G328200.1<br>Gorai.007G328500.1<br>Gorai.007G328600.1<br>Gorai.007G328800.1<br>Gorai.007G328900.1<br>Gorai.007G329100.1	TIR-NBS-LRR
HG21-01	Gorai.010G112800.1<br>Gorai.009G055800.1	Leucine-rich repeat receptor-like protein kinase family protein
HG21-02	Gorai.010G067400.1<br>Gorai.009G022300.1	Leucine-rich repeat receptor-like protein kinase family protein
HG21-03	Gorai.001G257700.1<br>Gorai.007G077900.1	Leucine-rich repeat receptor-like protein kinase family protein
HG21-04	Gorai.011G125200.1<br>Gorai.005G120900.1	Leucine-rich repeat receptor-like protein kinase family protein
HG21-05	Gorai.005G193500.1<br>Gorai.009G348000.1	Leucine-rich repeat receptor-like protein kinase family protein
HG22-01	Gorai.011G017800.1<br>Gorai.011G018100.1	CC-NBS-LRR
HG22-02	Gorai.011G018200.1<br>Gorai.011G018400.1	CC-NBS-LRR
HG22-03	Gorai.001G036800.1<br>Gorai.009G307500.1	Leucine-rich repeat receptor-like protein kinase family protein
HG22-04	Gorai.005G155800.1<br>Gorai.005G156000.1	CC-NBS-LRR
HG22-05	Gorai.010G145400.1<br>Gorai.010G221300.1<br>Gorai.010G145300.1	CC-NBS-LRR
HG22-06	Gorai.010G144800.1<br>Gorai.005G155900.1<br>Gorai.005G156100.1	CC-NBS-LRR
HG22-07	Gorai.006G084000.1<br>Gorai.006G084100.1	CC-NBS-LRR
HG22-08	Gorai.006G084500.1<br>Gorai.006G084600.1<br>Gorai.009G388400.1	CC-NBS-LRR

Sub-Homology groups	Gene ID	Functional annotation
HG23-01	Gorai.004G210200.1<br>Gorai.006G209800.1	Leucine-rich repeat receptor-like protein kinase family protein
HG24-01	Gorai.013G195600.1<br>Gorai.007G256600.1	Leucine-rich repeat receptor-like protein kinase family protein
HG24-02	Gorai.012G088400.1<br>Gorai.001G269200.1	Leucine-rich repeat receptor-like protein kinase family protein
HG28-01	Gorai.010G145800.1<br>Gorai.010G221000.1	CC-NBS-LRR
HG30-01	Gorai.007G330900.1<br>Gorai.007G365800.1<br>Gorai.007G366100.1<br>Gorai.007G366200.1<br>Gorai.007G366500.1	Receptor like protein<br>NB-ARC domain-containing disease resistance protein
HG30-02	Gorai.007G364100.1<br>Gorai.007G364400.1<br>Gorai.007G365900.1	Receptor like protein<br>NB-ARC domain-containing disease resistance protein
HG30-03	Gorai.007G361000.1<br>Gorai.007G361200.1	Receptor like protein
HG30-04	Gorai.009G439000.1<br>Gorai.009G439400.1<br>Gorai.009G439600.1<br>Gorai.009G439900.1	Receptor like protein<br>NB-ARC domain-containing disease resistance protein
HG30-05	Gorai.013G231500.1<br>Gorai.013G232000.1<br>Gorai.013G232100.1<br>Gorai.013G232200.1<br>Gorai.013G232300.1<br>Gorai.NG013600.1	Receptor like protein
HG30-06	Gorai.011G075600.1<br>Gorai.011G076300.1	Receptor like protein
HG30-07	Gorai.003G163400.1<br>Gorai.003G163700.1	Receptor like protein
HG30-08	Gorai.005G009300.1<br>Gorai.005G009700.1<br>Gorai.005G009900.1	NB-ARC domain-containing disease resistance protein
HG30-09	Gorai.010G013100.1<br>Gorai.NG019500.1	Receptor like protein
HG32-01	Gorai.010G160400.1<br>Gorai.011G038000.1	TIR-NBS-LRR
HG34-01	Gorai.003G032400.1<br>Gorai.008G086100.1<br>Gorai.007G226700.1	TIR-NBS-LRR
HG35-01	Gorai.002G048400.1<br>Gorai.002G048700.1	LRR and NB-ARC domains-containing disease resistance protein
HG40-01	Gorai.011G029600.1<br>Gorai.006G087900.1	TIR-NBS-LRR
HG42-01	Gorai.009G310300.1<br>Gorai.011G236200.1<br>Gorai.012G006200.1	Leucine-rich receptor-like protein kinase family protein

Note: Match rate and identities ⩾ 80%. For sub-homology groups, the identities and match rates are more than 80%. The sub-homology groups were classified according to the homology group clustering with identities ⩾ 30% and match rates ⩾ 33%.

Table S5 Information on Rgrcs in the *G. raimondii* genome

Cluster ID	Chromosome	Star	End	Length (kb)	Total genes	RGA genes	Ratio	RGA density (kb per gene)
Rgrc1	Chr02	1259105	1327255	68	11	9	81.82%	6.2
Rgrc2	Chr02	3676992	4709667	1033	86	29	33.72%	12.0
Rgrc3	Chr02	6992947	7182017	189	7	7	100.00%	27.0
Rgrc4	Chr02	62280505	62492332	211	26	7	26.92%	8.1
Rgrc5	Chr03	44340404	44594330	254	22	11	50.00%	11.5
Rgrc6	Chr05	2297846	2905491	608	60	21	35.00%	10.1
Rgrc7	Chr05	43721895	44029996	308	10	6	60.00%	30.8
Rgrc8	Chr06	4964525	5988138	1023	32	14	43.75%	32.0
Rgrc9	Chr06	31693476	31865926	172	8	7	87.50%	21.5
Rgrc10	Chr07	49603046	49917741	315	15	9	60.00%	21.0
Rgrc11	Chr07	53320902	56608929	3288	223	103	46.19%	14.7
Rgrc12	Chr07	58647174	58968563	321	20	9	45.00%	16.1
Rgrc13	Chr07	59223291	59944023	721	56	21	37.50%	12.9
Rgrc14	Chr09	48728294	52935043	4207	254	82	32.28%	16.6
Rgrc15	Chr09	68454601	69323105	869	62	19	30.65%	14.0
Rgrc16	Chr09	69883966	70039441	155	15	11	73.33%	10.3
Rgrc17	Chr10	3803868	3911385	108	15	7	46.67%	7.2
Rgrc18	Chr10	38052901	38503644	451	18	13	72.22%	25.1
Rgrc19	Chr10	59682531	60155580	473	55	19	34.55%	8.6
Rgrc20	Chr11	1202490	1282403	80	12	9	75.00%	6.7
Rgrc21	Chr11	52346514	52693064	347	19	7	36.84%	18.3
Rgrc22	Chr11	58795113	59139270	344	23	17	73.91%	15.0
Rgrc23	Chr11	59369171	59754486	385	30	16	53.33%	12.8
Rgrc24	Chr11	61230953	61780484	550	50	21	42.00%	11.0
Rgrc25	Chr11	62476142	62556393	80	10	7	70.00%	8.0
Rgrc26	Chr13	54994327	55093728	99	9	8	88.89%	11.0
Total Rgrcs				16659	1148	489	42.60%	14.5
Genome				737800	37505	1004	2.70%	19.7

Note: the average density of RGA genes in Rgrcs lower than genome is remarked in blue. The RGA ratio was calculated using the number of RGA genes compared with the total number of genes in the Rgrc. The RGA density was calculated by dividing the length of the Rgrc by the number of coding genes in the Rgrc.

Table S6 Statistical analysis of RGA genes in Rgrcs

Family ID	RGA gene type	Genes in Rgrcs	Ratio
R-I	CC-NBS-LRR	22	68.8%
R-II	Cysteine-rich RLK	34	56.7%
R-III	Disease resistance family protein / LRR family protein	27	58.7%
R-IV	Leucine-rich receptor-like protein kinase family protein	9	15.5%
R-V	Leucine-rich repeat protein kinase family protein	60	26.7%
R-VI	Leucine-rich repeat receptor-like protein kinase family protein	27	61.4%
R-VII	Leucine-rich repeat transmembrane protein kinase	26	33.3%
R-VIII	LRR and NB-ARC domains-containing disease resistance protein	44	55.7%
R-IX	NB-ARC domain-containing disease resistance protein	129	66.5%
R-X	Receptor like protein	78	54.2%
R-XI	TIR-NBS-LRR	33	75.0%

Table S7 Summary of sequencing yields and alignments

Sample	Total Reads	Total BasePairs	Total Mapped Reads	Perfect Match	<=2bp Mismatch	**Unique Match**	Multi-position Match	Total Unmapped Reads
CK	189,665,959	9,293,631,991	146,076,927	81,864,756	64,212,171	**136,078,729**	9,998,198	43,589,032
I2	206,241,051	10,105,811,499	158,248,670	87,782,188	70,466,482	**145,793,164**	12,455,506	47,992,381
I6	200,032,573	9,801,596,077	153,728,630	86,230,510	67,498,120	**139,666,811**	14,061,819	46,303,943
I2	197,486,498	9,676,838,402	151,781,769	85,072,331	66,709,438	**138,886,710**	12,895,059	45,704,729
I24	202,316,916	9,913,528,884	154,681,136	87,770,922	66,910,214	**139,929,427**	14,751,709	47,635,780
I48	199,002,295	9,751,112,455	151,800,776	85,231,800	66,568,976	**137,947,577**	13,853,199	47,201,519
I72	195,711,304	9,589,853,896	149,424,002	83,543,354	65,880,648	**136,460,406**	12,963,596	46,287,302

Note: ‘CK’ is the mock-inoculated sample, ‘I2–I72’ represents the six inoculation time points.

Table S8 Statistical analysis of DEGs in the *G. raimondii* genome and its RGA gene set

	FDR<0.001, *p* -value<0.001	FDR<0.001, *p* -value<0.001, $\log_2$Ratio≥|1.0|	FDR<0.001, *p* -value<0.001, $\log_2$Ratio≥|2.0|
Coding sequence of *G. ranmondii*			
Total DEGs	28360	17517	8122
DEGs in common	13229	9811	5106
RGA genes set			
Total DEGs	723	585	294
DEGs in common	319	285	168

Table S10 Statistical analysis of DEGs in the 11 RGA gene families

**Family**	Total	**I2(U)**	**I2(D)**	**I6(U)**	**I6(D)**	**I12(U)**	**I12(D)**	**I24(U)**	**I24(D)**	**I48(U)**	**I48(D)**	**I72(U)**	**I72(D)**	**Common**
CC-NBS-LRR	32	12(10)	11(5)	11(8)	10(8)	13(7)	9(6)	12(7)	10(8)	12(9)	11(5)	11(7)	9(3)	**13(4)**
Cysteine-rich RLK	60	22(18)	10(4)	24(19)	13(9)	25(21)	12(4)	27(24)	15(11)	27(24)	12(11)	27(24)	13(10)	**24(15)**
Disease resistance family protein_LRR family protein	46	13(11)	3(2)	10(9)	6(6)	9(9)	3(2)	10(10)	7(7)	14(12)	4(4)	14(11)	4(3)	**8(6)**
Leucine-rich receptor-like protein kinase family protein	58	19(13)	16(8)	27(15)	10(7)	26(14)	8(6)	31(20)	9(7)	34(31)	7(3)	30(25)	8(6)	**25(10)**
Leucine-rich repeat protein kinase family protein	225	50(31)	75(49)	51(30)	73(49)	56(28)	73(43)	64(39)	76(53)	79(50)	55(37)	69(38)	63(43)	**73(28)**
Leucine-rich repeat receptor-like protein kinase family protein	44	11(8)	8(6)	8(7)	9(8)	7(5)	8(7)	8(6)	9(9)	8(7)	9(7)	8(6)	7(7)	**11(7)**
Leucine-rich repeat transmembrane protein kinase	78	33(26)	17(9)	38(25)	15(9)	33(27)	14(8)	38(24)	12(8)	43(29)	7(5)	35(21)	11(8)	**34(17)**
LRR and NB-ARC domains-containing disease resistance protein	79	25(9)	18(7)	28(11)	20(8)	28(11)	12(4)	34(18)	17(9)	35(21)	16(7)	29(16)	11(3)	**22(6)**
NB-ARC domain-containing disease resistance protein	194	70(43)	28(10)	79(49)	28(7)	81(45)	22(5)	93(57)	23(9)	107(69)	22(10)	103(55)	15(5)	**68(30)**
Receptor like protein	144	14(11)	24(16)	18(14)	37(31)	12(10)	25(21)	15(11)	42(35)	23(18)	34(29)	23(12)	24(23)	**20(11)**
TIR-NBS-LRR	44	19(9)	11(4)	17(9)	12(7)	17(6)	9(5)	16(7)	12(7)	15(7)	18(11)	16(7)	15(12)	**15(6)**
**Total DEG in RGA genes**	1004	**288 (189)**	**221 (120)**	**311 (196)**	**233 (149)**	**307 (183)**	**195 (111)**	**348 (223)**	**232 (163)**	**397 (277)**	**195 (129)**	**365 (222)**	**180 (123)**	**313 (140)**

Note: FDR<0.001, p-value<0.001, log2Ratio≥|1.0|, and the number in bracket statistic by log2Ratio≥|2.0|. I2(U), the up-regulated DEGs of cotton at two hours after inoculation.

Table S11 Information regarding differentially expressed RGA genes involved in the plant-pathogen interaction pathway

Resistance gene name	KEGG Orthology ID	DEGs in RGA genes
BAK1	K13416	29
SERK4	K13417	5
FLS2	K13420	121
EFR	K13428	39
PBS5	K13430	22
RPM1	K13457	129
RPS2	K13459	84
RPS5	K13460	22
Total	-	451

Note: FDR<0.001 and *p*- value<0.001

Table S12 Statistical analysis of potential DEGs in *G. barbadense* in response to V. dahliae

Family ID	Family description	DEG[*]	% in family[#]	In VdRL loci
R-I	CC-NBS-LRR	2	6.3	1
R-II	Cysteine-rich RLK	21	35.0	15
R-III	Disease resistance family protein / LRR family protein	14	30.4	5
R-IV	Leucine-rich receptor-like protein kinase family protein	21	36.2	1
R-V	Leucine-rich repeat protein kinase family protein	36	16.0	7
R-VI	Leucine-rich repeat receptor-like protein kinase family protein	9	20.5	4
R-VII	Leucine-rich repeat transmembrane protein kinase	21	26.9	9
R-VIII	LRR and NB-ARC domains-containing disease resistance protein	5	6.3	1
R-IX	NB-ARC domain-containing disease resistance protein	24	12.4	15
R-X	Receptor like protein	13	9.0	3
R-XI	TIR-NBS-LRR	2	4.5	2
Total		168	-	63

*DEG: FDR<0.001, *p*-value<0.001, log_2Ratio≥|2.0|, and at least one inoculation time point is up-regulated. [#]Proportion of potential DEGs in their RGA gene family

Table S13 Potential DEGs and VdRLs in *G. barbadense* in response to V. dahliae

Gene-ID	VdRL	Cluster	Family-ID	Family description	HG	I2/CK	I6/CK	I12/CK	I24/CK	I48/CK	I72/CK
Gorai.001G016300.1			R-V	Leucine-rich repeat protein kinase family protein		2.57	2.43	2.72	1.53	1.50	0.83
Gorai.001G019800.1			R-IV	Leucine-rich receptor-like protein kinase family protein	HG17-27	2.30	2.01	2.47	2.11	2.57	0.53
Gorai.001G032100.1			R-V	Leucine-rich repeat protein kinase family protein		-0.24	0.89	-0.05	1.27	3.16	2.00
Gorai.001G223400.1			R-X	receptor like protein		-1.42	1.36	1.01	2.14	1.80	1.45
Gorai.002G048300.1	VdRL01	Rgrc2	R-VII	Leucine-rich repeat transmembrane protein kinase	HG17-12	1.58	1.83	1.99	2.26	1.91	1.53
Gorai.002G048600.1		Rgrc2	R-VII	Leucine-rich repeat transmembrane protein kinase	HG17-12	1.34	1.90	2.08	1.99	1.37	1.00
Gorai.002G048800.1		Rgrc2	R-VII	Leucine-rich repeat transmembrane protein kinase	HG17-12	1.73	1.90	2.26	2.38	2.22	1.05
Gorai.002G048900.1		Rgrc2	R-VII	Leucine-rich repeat transmembrane protein kinase		2.23	1.63	1.58	0.25	0.36	-0.44
Gorai.002G051100.1	VdRL02	Rgrc2	R-V	Leucine-rich repeat protein kinase family protein	HG17-14	1.21	0.54	-0.46	1.27	2.43	1.70
Gorai.002G051400.1		Rgrc2	R-V	Leucine-rich repeat protein kinase family protein		1.47	-0.05	-0.05	0.94	3.97	3.00
Gorai.002G051900.1		Rgrc2	R-V	Leucine-rich repeat protein kinase family protein	HG17-14	-0.70	-0.31	-2.05	0.06	2.20	1.33
Gorai.002G064300.1			R-V	Leucine-rich repeat protein kinase family protein		3.19	2.79	2.36	0.35	-0.38	-1.17
Gorai.002G079000.1			R-I	CC-NBS-LRR		1.78	1.98	2.01	2.19	2.00	1.91
Gorai.002G103300.1			R-IV	Leucine-rich receptor-like protein kinase family protein	HG17-16	-0.17	0.43	0.78	2.07	3.10	2.01
Gorai.002G105800.1			R-VII	Leucine-rich repeat transmembrane protein kinase		2.01	1.76	1.73	1.40	0.97	0.98
Gorai.002G208100.1			R-IX	NB-ARC domain-containing disease resistance protein		1.30	2.88	2.13	1.79	1.28	0.56
Gorai.002G217000.1			R-IV	Leucine-rich receptor-like protein kinase family protein	HG17-19	2.13	2.03	1.74	1.26	2.01	1.69
Gorai.002G229000.1			R-V	Leucine-rich repeat protein kinase family protein		0.23	0.88	0.44	0.77	2.07	1.63
Gorai.002G262300.1	VdRL03	Rgrc4	R-X	receptor like protein		2.59	3.55	3.35	1.99	-0.16	0.40
Gorai.003G011000.1			R-IV	Leucine-rich receptor-like protein kinase family protein		-0.58	0.21	0.18	0.70	2.02	1.56
Gorai.003G055300.1			R-III	disease resistance family protein_LRR family protein		2.50	2.66	2.63	2.62	2.88	3.33
Gorai.003G082600.1			R-V	Leucine-rich repeat protein kinase family protein		1.85	1.70	1.94	2.58	2.44	2.32
Gorai.003G148900.1			R-IX	NB-ARC domain-containing disease resistance protein		5.05	7.94	5.98	5.66	4.33	3.53
Gorai.003G172400.1	VdRL04	Rgrc5	R-XI	TIR-NBS-LRR	HG01-01	2.18	2.26	1.54	0.28	-0.60	-0.78
Gorai.004G023400.1			R-X	receptor like protein		0.69	2.71	2.86	3.85	4.14	4.23
Gorai.004G024700.1			R-V	Leucine-rich repeat protein kinase family protein		2.40	-0.23	0.35	0.34	0.79	-0.18
Gorai.004G045000.1			R-II	cysteine-rich RLK		3.48	4.11	3.82	3.16	1.92	1.74
Gorai.004G053700.1			R-X	receptor like protein		3.55	4.07	3.72	3.59	3.95	3.22
Gorai.004G054100.1			R-X	receptor like protein		3.63	4.31	3.50	3.78	4.22	3.25
Gorai.004G120000.1			R-VII	Leucine-rich repeat transmembrane protein kinase		0.99	0.91	1.10	1.71	2.46	1.95
Gorai.004G169400.1			R-VIII	LRR and NB-ARC domains-containing disease resistance protein		0.78	1.49	1.76	2.00	2.08	1.84

Gene-ID	VdRL	Cluster	Family-ID	Family description	HG	I2/CK	I6/CK	I12/CK	I24/CK	I48/CK	I72/CK
Gorai.004G169900.1			R-VIII	LRR and NB-ARC domains-containing disease resistance protein		1.06	2.12	1.28	2.94	4.02	3.33
Gorai.004G170000.1			R-IX	NB-ARC domain-containing disease resistance protein		0.29	1.21	1.26	2.83	3.52	3.26
Gorai.004G202400.1			R-V	Leucine-rich repeat protein kinase family protein		2.46	2.71	1.90	1.96	2.29	2.04
Gorai.004G204100.1			R-V	Leucine-rich repeat protein kinase family protein	HG17-24	2.39	3.69	3.73	3.48	2.39	2.09
Gorai.004G248900.1			R-III	disease resistance family protein_LRR family protein		2.76	3.33	3.53	3.07	2.20	2.09
Gorai.004G249900.1			R-V	Leucine-rich repeat protein kinase family protein	HG17-26	4.23	4.70	4.83	4.72	4.22	3.55
Gorai.004G250200.1			R-V	Leucine-rich repeat protein kinase family protein		0.00	0.35	0.67	1.73	2.06	1.79
Gorai.005G039600.1			R-VI	Leucine-rich repeat receptor-like protein kinase family protein	HG17-34	4.00	5.31	5.05	5.18	4.56	4.36
Gorai.005G054700.1			R-VI	Leucine-rich repeat receptor-like protein kinase family protein		-0.11	0.21	0.52	1.16	2.06	1.63
Gorai.005G078900.1			R-IV	Leucine-rich receptor-like protein kinase family protein		1.41	0.86	1.71	1.47	2.22	1.86
Gorai.005G097600.1			R-IV	Leucine-rich receptor-like protein kinase family protein		2.16	1.24	1.82	1.33	2.01	1.40
Gorai.005G135700.1			R-VII	Leucine-rich repeat transmembrane protein kinase		1.79	2.92	2.53	2.94	2.73	2.79
Gorai.005G155700.1	VdRL05	Rgrc7	R-II	cysteine-rich RLK		0.73	1.77	1.54	2.04	2.85	1.91
Gorai.005G155900.1		Rgrc7	R-II	cysteine-rich RLK	HG22-06	2.09	2.48	2.39	2.75	2.65	2.61
Gorai.005G156000.1		Rgrc7	R-II	cysteine-rich RLK	HG22-04	1.81	2.11	1.52	1.79	1.94	1.73
Gorai.005G156100.1		Rgrc7	R-II	cysteine-rich RLK	HG22-06	2.36	2.84	2.50	3.25	3.41	2.95
Gorai.005G265400.1			R-IV	Leucine-rich receptor-like protein kinase family protein	HG17-19	0.33	0.68	0.60	0.84	2.06	1.52
Gorai.005G268400.1			R-IV	Leucine-rich receptor-like protein kinase family protein		0.56	0.98	0.87	1.14	2.16	1.95
Gorai.006G040500.1			R-IX	NB-ARC domain-containing disease resistance protein		1.42	1.56	1.16	1.65	2.17	2.04
Gorai.006G082700.1			R-II	cysteine-rich RLK		2.38	3.60	3.11	3.03	2.61	3.05
Gorai.006G084300.1	VdRL06	Rgrc9	R-II	cysteine-rich RLK		1.73	1.45	1.94	1.80	2.26	2.16
Gorai.006G084400.1		Rgrc9	R-II	cysteine-rich RLK		-0.19	-0.97	-0.59	0.50	2.34	1.76
Gorai.006G084600.1		Rgrc9	R-II	cysteine-rich RLK	HG22-05	0.69	1.70	2.17	2.08	1.51	1.35
Gorai.006G137100.1			R-V	Leucine-rich repeat protein kinase family protein	HG17-23	0.06	1.32	1.20	2.17	2.98	2.10
Gorai.006G139200.1			R-VI	Leucine-rich repeat receptor-like protein kinase family protein		0.44	2.19	1.93	2.28	2.87	2.18
Gorai.006G219400.1			R-V	Leucine-rich repeat protein kinase family protein	HG17-34	2.75	3.11	3.26	2.87	2.46	2.36
Gorai.006G227000.1			R-IV	Leucine-rich receptor-like protein kinase family protein		-0.12	1.60	1.77	3.36	4.01	3.10
Gorai.007G062800.1			R-IV	Leucine-rich receptor-like protein kinase family protein	HG17-18	0.92	1.08	1.14	1.69	1.98	2.02
Gorai.007G079500.1			R-III	disease resistance family protein_LRR family protein		3.91	5.16	5.25	5.44	5.08	5.06
Gorai.007G256600.1			R-V	Leucine-rich repeat protein kinase family protein	HG24-01	-0.48	0.19	0.62	1.50	2.25	1.77
Gorai.007G273700.1			R-IV	Leucine-rich receptor-like protein kinase family protein		0.01	-1.27	-0.43	0.57	2.22	1.18
Gorai.007G280700.1			R-III	disease resistance family protein_LRR family protein		3.59	4.76	4.41	5.07	5.02	5.17

Gene-ID	VdRL	Cluster	Family-ID	Family description	HG	I2/CK	I6/CK	I12/CK	I24/CK	I48/CK	I72/CK
Gorai.007G281100.1			R-X	receptor like protein		0.89	2.00	0.85	1.09	1.56	0.20
Gorai.007G282300.1			R-X	receptor like protein		1.26	1.96	0.93	1.91	2.03	1.09
Gorai.007G290400.1		Rgrc10	R-I	CC-NBS-LRR		2.55	1.83	1.95	1.37	1.18	1.19
Gorai.007G290600.1	VdRL07	Rgrc10	R-IX	NB-ARC domain-containing disease resistance protein		1.47	1.19	1.90	2.28	2.32	2.65
Gorai.007G290700.1		Rgrc10	R-IX	NB-ARC domain-containing disease resistance protein		1.42	1.67	1.37	1.80	2.16	2.29
Gorai.007G295800.1			R-III	disease resistance family protein_LRR family protein		2.52	2.32	2.19	1.67	1.68	1.51
Gorai.007G323100.1	VdRL08	Rgrc11	R-IX	NB-ARC domain-containing disease resistance protein		1.90	1.92	1.54	2.20	2.39	2.25
Gorai.007G324300.1		Rgrc11	R-IX	NB-ARC domain-containing disease resistance protein	HG05-04	3.66	3.66	3.45	3.36	3.05	3.00
Gorai.007G329900.1		Rgrc11	R-VII	Leucine-rich repeat transmembrane protein kinase		2.58	2.92	2.59	2.54	1.92	1.40
Gorai.007G330000.1		Rgrc11	R-VII	Leucine-rich repeat transmembrane protein kinase		2.89	3.10	2.65	2.40	1.80	1.38
Gorai.007G330300.1	VdRL09	Rgrc11	R-VII	Leucine-rich repeat transmembrane protein kinase	HG17-38	2.36	2.74	2.44	2.46	2.05	1.83
Gorai.007G330500.1		Rgrc11	R-VII	Leucine-rich repeat transmembrane protein kinase		3.11	3.38	2.81	2.44	1.90	1.51
Gorai.007G330700.1		Rgrc11	R-VII	Leucine-rich repeat transmembrane protein kinase	HG17-38	3.24	3.41	2.88	2.42	2.12	1.74
Gorai.007G336000.1		Rgrc11	R-III	disease resistance family protein_LRR family protein	HG20-01	0.89	0.95	0.95	-0.06	2.71	1.88
Gorai.007G338900.1		Rgrc11	R-III	disease resistance family protein_LRR family protein		2.69	0.95	2.86	3.59	3.78	5.11
Gorai.007G339300.1		Rgrc11	R-III	disease resistance family protein_LRR family protein	HG20-02	1.38	1.16	1.38	2.44	4.07	4.11
Gorai.007G339700.1	VdRL10	Rgrc11	R-III	disease resistance family protein_LRR family protein		0.21	-1.05	0.95	0.94	3.14	2.33
Gorai.007G339900.1		Rgrc11	R-III	disease resistance family protein_LRR family protein	HG20-02	1.76	-0.22	1.18	0.47	1.66	2.33
Gorai.007G340100.1		Rgrc11	R-III	disease resistance family protein_LRR family protein		3.13	3.83	2.91	2.85	2.14	2.46
Gorai.007G357700.1	VdRL11	Rgrc12	R-IX	NB-ARC domain-containing disease resistance protein		1.33	2.11	1.83	1.84	1.69	1.70
Gorai.007G361300.1	VdRL12	Rgrc13	R-IX	NB-ARC domain-containing disease resistance protein		1.69	3.27	0.95	2.27	2.56	3.00
Gorai.007G364700.1	VdRL13	Rgrc13	R-IX	NB-ARC domain-containing disease resistance protein		2.56	1.97	2.00	2.55	2.63	2.09
Gorai.007G364900.1		Rgrc13	R-IX	NB-ARC domain-containing disease resistance protein		1.53	1.16	1.44	2.11	2.64	2.17
Gorai.008G023200.1			R-V	Leucine-rich repeat protein kinase family protein		-1.70	-0.11	0.26	1.64	2.18	1.15
Gorai.008G045400.1			R-IV	Leucine-rich receptor-like protein kinase family protein	HG17-19	1.78	3.43	2.96	4.06	4.17	4.39
Gorai.008G086100.1			R-X	receptor like protein	HG34-01	-0.79	0.61	-0.21	0.84	2.64	1.92
Gorai.009G007000.1			R-IX	NB-ARC domain-containing disease resistance protein		3.39	1.79	2.41	1.66	1.75	1.55
Gorai.009G010400.1			R-X	receptor like protein		2.89	2.81	1.95	1.27	1.30	1.70
Gorai.009G033000.1			R-IX	NB-ARC domain-containing disease resistance protein		2.17	3.05	3.02	2.50	2.36	3.39
Gorai.009G055800.1			R-V	Leucine-rich repeat protein kinase family protein	HG21-01	0.65	1.13	1.20	1.59	2.00	1.51
Gorai.009G102800.1			R-VII	Leucine-rich repeat transmembrane protein kinase		3.92	5.15	4.79	3.62	3.49	3.16
Gorai.009G119100.1			R-II	cysteine-rich RLK		0.62	1.03	1.13	1.91	2.42	2.19

Gene-ID	VdRL	Cluster	Family-ID	Family description	HG	I2/CK	I6/CK	I12/CK	I24/CK	I48/CK	I72/CK
Gorai.009G207700.1			R-IV	Leucine-rich receptor-like protein kinase family protein		1.17	1.39	1.50	1.85	2.30	2.14
Gorai.009G237700.1			R-VI	Leucine-rich repeat receptor-like protein kinase family protein	HG17-06	1.95	1.48	2.18	1.39	1.96	1.66
Gorai.009G286600.1			R-IV	Leucine-rich receptor-like protein kinase family protein		0.39	1.42	0.45	1.47	2.86	2.28
Gorai.009G287800.1			R-IV	Leucine-rich receptor-like protein kinase family protein		0.81	1.19	1.11	1.56	2.09	1.80
Gorai.009G306900.1			R-V	Leucine-rich repeat protein kinase family protein	HG17-07	4.61	3.73	4.15	2.19	3.01	2.05
Gorai.009G307100.1			R-V	Leucine-rich repeat protein kinase family protein	HG17-07	5.16	4.36	4.65	3.15	4.00	2.70
Gorai.009G315400.1			R-IX	NB-ARC domain-containing disease resistance protein		2.40	2.31	1.76	2.09	2.73	1.99
Gorai.009G315500.1			R-VIII	LRR and NB-ARC domains-containing disease resistance protein		2.39	2.08	1.94	2.39	2.72	2.25
Gorai.009G336500.1			R-II	cysteine-rich RLK		-0.92	-0.86	-0.68	1.63	3.91	3.16
Gorai.009G348000.1			R-V	Leucine-rich repeat protein kinase family protein	HG21-05	1.12	1.45	1.30	2.00	2.15	1.92
Gorai.009G354600.1			R-X	receptor like protein		0.89	0.80	1.95	1.08	2.56	2.14
Gorai.009G363600.1			R-VII	Leucine-rich repeat transmembrane protein kinase	HG17-13	2.05	2.96	2.46	2.07	1.27	1.35
Gorai.009G370400.1	VdRL14	Rgrc14	R-IV	Leucine-rich receptor-like protein kinase family protein		0.25	-0.53	0.15	0.94	2.25	1.00
Gorai.009G379500.1	VdRL15	Rgrc14	R-IX	NB-ARC domain-containing disease resistance protein		4.41	4.41	4.20	4.64	4.73	4.25
Gorai.009G379800.1		Rgrc14	R-IX	NB-ARC domain-containing disease resistance protein	HG01-05	1.75	2.97	3.42	3.50	2.66	2.28
Gorai.009G380300.1		Rgrc14	R-IX	NB-ARC domain-containing disease resistance protein		0.51	0.77	1.11	1.53	2.36	2.26
Gorai.009G380600.1		Rgrc14	R-VIII	LRR and NB-ARC domains-containing disease resistance protein	HG12-01	1.39	1.89	1.37	2.06	1.97	2.17
Gorai.009G385800.1	VdRL16	Rgrc14	R-IX	NB-ARC domain-containing disease resistance protein	HG01-05	0.98	1.13	0.95	1.48	2.12	1.74
Gorai.009G388400.1	VdRL17	Rgrc14	R-II	cysteine-rich RLK	HG22-08	0.69	1.89	1.99	2.67	2.53	2.11
Gorai.009G434100.1			R-VIII	LRR and NB-ARC domains-containing disease resistance protein	HG12-02	0.62	0.59	1.54	1.73	2.26	2.19
Gorai.009G437000.1	VdRL18	Rgrc15	R-IX	NB-ARC domain-containing disease resistance protein		1.98	2.12	1.76	2.12	2.30	2.39
Gorai.009G449100.1	VdRL19	Rgrc16	R-X	receptor like protein		1.25	1.87	1.88	2.37	2.39	2.87
Gorai.009G452000.1			R-IV	Leucine-rich receptor-like protein kinase family protein	HG17-16	3.64	4.02	3.72	4.22	4.72	3.91
Gorai.010G030500.1			R-VII	Leucine-rich repeat transmembrane protein kinase		1.15	0.58	0.67	1.41	2.42	1.61
Gorai.010G040600.1	VdRL20	Rgrc17	R-VI	Leucine-rich repeat receptor-like protein kinase family protein		2.02	0.04	0.75	-2.38	-1.93	-1.32
Gorai.010G040900.1		Rgrc17	R-VI	Leucine-rich repeat receptor-like protein kinase family protein	HG17-04	2.87	1.43	1.54	0.36	0.39	0.00
Gorai.010G041100.1		Rgrc17	R-VI	Leucine-rich repeat receptor-like protein kinase family protein	HG19-03	3.39	1.87	2.19		-0.11	0.51
Gorai.010G041500.1		Rgrc17	R-VI	Leucine-rich repeat receptor-like protein kinase family protein		2.21	0.73	1.05	-0.54	0.17	-1.80
Gorai.010G112800.1			R-V	Leucine-rich repeat protein kinase family protein	HG21-01	1.33	1.63	2.04	2.17	2.64	2.58
Gorai.010G136200.1			R-IV	Leucine-rich receptor-like protein kinase family protein		-1.34	-0.44	0.21	2.07	3.09	1.64

Gene-ID	VdRL	Cluster	Family-ID	Family description	HG	I2/CK	I6/CK	I12/CK	I24/CK	I48/CK	I72/CK
Gorai.010G144600.1		Rgrc18	R-II	cysteine-rich RLK		2.10	2.30	2.02	2.84	2.58	2.79
Gorai.010G144800.1	VdRL21	Rgrc18	R-II	cysteine-rich RLK	HG22-06	2.35	2.46	2.41	2.87	2.70	2.71
Gorai.010G145100.1		Rgrc18	R-II	cysteine-rich RLK		1.69	2.34	2.04	2.64	3.47	2.70
Gorai.010G146300.1		Rgrc18	R-II	cysteine-rich RLK		1.82	2.26	2.85	2.66	1.79	2.42
Gorai.010G165300.1			R-IV	Leucine-rich receptor-like protein kinase family protein		4.43	3.54	3.63	1.28	1.81	1.56
Gorai.010G176100.1			R-II	cysteine-rich RLK		1.28	-0.17	1.95	1.85	2.64	2.25
Gorai.010G219700.1			R-V	Leucine-rich repeat protein kinase family protein		-0.16	0.36	0.25	0.80	2.10	1.17
Gorai.010G221300.1			R-II	cysteine-rich RLK	HG22-05	2.27	1.34	2.21	0.41	1.36	0.39
Gorai.010G228900.1	VdRL22	Rgrc19	R-X	receptor like protein		2.45	2.78	2.13	1.28	0.55	2.04
Gorai.010G230800.1			R-V	Leucine-rich repeat protein kinase family protein		0.77	0.71	0.90	1.67	2.42	2.04
Gorai.011G001200.1			R-IV	Leucine-rich receptor-like protein kinase family protein		1.38	1.54	1.56	1.98	2.35	2.22
Gorai.011G001900.1			R-V	Leucine-rich repeat protein kinase family protein		1.21	0.54	2.41	4.67	4.18	3.17
Gorai.011G004900.1			R-IV	Leucine-rich receptor-like protein kinase family protein		-0.68	0.36	0.44	1.26	2.78	1.94
Gorai.011G017300.1		Rgrc20	R-IX	NB-ARC domain-containing disease resistance protein		0.04	2.43		3.10	4.13	3.96
Gorai.011G017600.1	VdRL23	Rgrc20	R-II	cysteine-rich RLK		3.21	2.26	2.50	2.27	2.04	1.64
Gorai.011G017700.1		Rgrc20	R-II	cysteine-rich RLK		2.94	1.36	1.97	0.46	0.39	0.02
Gorai.011G018000.1		Rgrc20	R-II	cysteine-rich RLK		1.86	2.07	1.73	1.63	0.98	1.09
Gorai.011G052000.1			R-VII	Leucine-rich repeat transmembrane protein kinase	HG17-32	2.64	3.00	3.52	3.34	3.23	2.54
Gorai.011G052100.1			R-VII	Leucine-rich repeat transmembrane protein kinase	HG17-32	3.16	3.62	3.98	3.91	3.78	2.93
Gorai.011G052300.1			R-VII	Leucine-rich repeat transmembrane protein kinase	HG17-30	2.13	1.74	1.62	0.67	0.22	0.06
Gorai.011G052400.1			R-VII	Leucine-rich repeat transmembrane protein kinase	HG17-31	2.55	2.23	2.05	0.80	0.57	0.05
Gorai.011G125500.1			R-V	Leucine-rich repeat protein kinase family protein		1.09	2.95	2.80	3.78	3.64	3.08
Gorai.011G133300.1			R-IX	NB-ARC domain-containing disease resistance protein		1.19	1.64	1.44	2.20	2.40	1.99
Gorai.011G143400.1			R-III	disease resistance family protein_LRR family protein	HG20-05	4.27	2.58	3.42	1.90	2.17	0.90
Gorai.011G143500.1			R-III	disease resistance family protein_LRR family protein	HG20-05	4.32	2.51	3.49	2.14	2.24	1.00
Gorai.011G162100.1			R-VI	Leucine-rich repeat receptor-like protein kinase family protein		-0.95	0.14	-0.34	0.59	2.91	1.85
Gorai.011G177700.1			R-III	disease resistance family protein_LRR family protein		-1.44	0.21	-0.05	0.94	2.30	1.14
Gorai.011G257100.1			R-X	receptor like protein		3.62	3.61	4.39	5.19	6.25	5.08
Gorai.011G258700.1		Rgrc22	R-V	Leucine-rich repeat protein kinase family protein	HG17-02	4.44	3.07	3.67	2.75	2.52	2.42
Gorai.011G259300.1	VdRL24	Rgrc22	R-V	Leucine-rich repeat protein kinase family protein		2.01	0.74	1.47	0.24	0.16	-0.13
Gorai.011G260300.1		Rgrc22	R-V	Leucine-rich repeat protein kinase family protein	HG17-02	-0.53	1.65	-0.46	2.40	0.56	1.46
Gorai.011G270000.1			R-IX	NB-ARC domain-containing disease resistance protein		2.56	2.53	2.35	2.28	2.30	1.65

Gene-ID	VdRL	Cluster	Family-ID	Family description	HG	I2/CK	I6/CK	I12/CK	I24/CK	I48/CK	I72/CK
Gorai.011G282200.1	VdRL25	Rgrc24	R-XI	TIR-NBS-LRR		1.73	1.98	1.87	2.09	2.03	1.78
Gorai.011G285500.1		Rgrc24	R-V	Leucine-rich repeat protein kinase family protein		1.02	1.56	1.97	2.87	2.50	2.26
Gorai.012G106100.1			R-V	Leucine-rich repeat protein kinase family protein		4.01	4.90	4.36	3.42	3.01	2.88
Gorai.013G036900.1			R-V	Leucine-rich repeat protein kinase family protein	HG17-03	2.59	2.81	2.55	2.12	1.81	1.91
Gorai.013G047000.1			R-V	Leucine-rich repeat protein kinase family protein		-1.36	-0.93	-0.10	1.40	2.22	1.56
Gorai.013G062400.1			R-V	Leucine-rich repeat protein kinase family protein		1.32	2.26	0.39	0.79	1.14	-0.15
Gorai.013G156100.1			R-V	Leucine-rich repeat protein kinase family protein		2.06	2.65	2.54	4.03	3.93	4.05
Gorai.013G232300.1	VdRL26	Rgrc26	R-IX	NB-ARC domain-containing disease resistance protein	HG03-05	2.11	2.12	1.89	1.83	1.80	1.35
Gorai.N011100.1			R-VII	Leucine-rich repeat transmembrane protein kinase		2.99	2.30	2.43	2.53	2.93	2.66
Gorai.N011200.1			R-VII	Leucine-rich repeat transmembrane protein kinase		4.19	4.80	4.47	4.48	4.01	3.20
Gorai.N016200.1			R-V	Leucine-rich repeat protein kinase family protein		3.89	1.54	2.95	1.94	2.56	2.59

*DEG: FDR<0.001, *p*-value<0.001, $\log_2$Ratio≥|2.0|, and at least one inoculation time point is up-regulated; VdRL: *V. dahliae* Response Loci; HG: homology group, chultered with identities and match rated up to 80%, respectively. I2/CK, gene expression analysis in the sample two hours after inoculation (I2) compared to the control sample (CK). Gorai.007G336000.1 do not classified in to VdRL10 for the long physical distance

Table S14 Information regarding VdRLs

VdRL	Chrosome	In Cluster	Start#	End#	Locus length	RGA genes*	Adjacent to QTL	Distance (Mb)	p-value	Rsq_marker$	Enviroment
VdRL01	Chr02	Rgrc2	4086173	4189409	103236	4	/	/	/	/	/
VdRL02	Chr02	Rgrc2	4410273	4566337	156064	3	/	/	/	/	/
VdRL03	Chr02	Rgrc4	62260505	62304265	43760	1	/	/	/	/	/
VdRL04	Chr03	Rgrc5	44320404	44366582	46178	1	NAU5233	1.99	0.0057	0.0287	disease nursery
VdRL05	Chr03	Rgrc7	43863446	44049996	186550	4	/	/	/	/	/
**VdRL06**	**Chr06**	**Rgrc9**	**31750489**	**31885926**	**135437**	**3**	**NAU2753**	**0.69**	**10.26(LOD score)**	**0.168**	**greenhouse**
**VdRL07**	**Chr07**	**Rgrc10**	**49749827**	**49884838**	**135011**	**3**	**MUCS219**	**0.82**	**7.92(LOD score)**	**0.157**	**greenhouse**
VdRL08	Chr07	Rgrc11	54093871	54356747	262876	2	NAU5428	3.66	6.68E-04	0.1093	disease nursery
VdRL09	Chr07	Rgrc11	55194116	55327259	133143	5	NAU5428	2.56	6.68E-04	0.1093	disease nursery
VdRL10	Chr07	Rgrc11	56393994	56552744	158750	5	NAU5428	1.36	6.68E-04	0.1093	disease nursery
**VdRL11**	**Chr07**	**Rgrc12**	**58940505**	**58984982**	**44477**	**1**	**CIR196**	**0.73**	**0.0064**	**0.00684**	**disease nursery**
VdRL12	Chr07	Rgrc13	59296102	59337374	41272	1	CIR196	1.09	0.0064	0.00684	disease nursery
VdRL13	Chr07	Rgrc13	59655479	59727701	72222	2	CIR196	1.45	0.0064	0.00684	disease nursery
VdRL14	Chr09	Rgrc14	49799003	49840473	41470	1	/	/	/	/	/
VdRL15	Chr09	Rgrc14	51449320	51685831	236511	4	/	/	/	/	/
VdRL16	Chr09	Rgrc14	52341417	52384884	43467	1	/	/	/	/	/
VdRL17	Chr09	Rgrc14	52590349	52633316	42967	1	/	/	/	/	/
**VdRL18**	**Chr09**	**Rgrc15**	**68726929**	**68772363**	**45434**	**1**	**NAU3592**	**0.44**	**0.0057**	**0.0488**	**disease nursery**
**VdRL19**	**Chr09**	**Rgrc16**	**70016505**	**70059441**	**42936**	**1**	**NAU3592**	**0.85**	**0.0057**	**0.0488**	**disease nursery**
VdRL20	Chr10	Rgrc17	3806646	3900232	93586	4	BNL827	2.81	NS	NS	NS
VdRL21	Chr10	Rgrc18	38032901	38523644	490743	4	/	/	/	/	/
VdRL22	Chr10	Rgrc19	60046011	60089717	43706	1	/	/	/	/	/
VdRL23	Chr11	Rgrc20	1182490	1270413	87923	4	/	/	/	/	/
VdRL24	Chr11	Rgrc22	58846679	59159270	312591	3	BNL3646	2.95	0.0465	0.0317	greenhouse
**VdRL25**	**Chr11**	**Rgrc24**	**61406779**	**61800484**	**393705**	**2**	**BNL3646**	**0.39**	**0.0465**	**0.0317**	**greenhouse**
VdRL26	Chr13	Rgrc26	55070901	55113728	42827	1	/	/	/	/	/

Loci position was extended 20 kb according to the star and end RGA genes; * FDR<0.001, *p*-value<0.001, $\log_2$Ratio⩾|2.0|, and at least one inoculation time point is up-regulated. The distance between VdRLs and the known Verticillium wilt resistance QTL less than 1-Mb are in a bold font. The physical distance between NAU5428 and VdRL08 is more than 3-Mb, marker in gray; $total explained phenotypic variation in previous research, the datas were collected from the reference 28,81 and 95, ; NS, not statistically significant. The Verticillium wilt resistance QTLs were reported in prrevious research [33-37].

Table S15 The RGA genes family enrichment in VdRLs

Family-ID	RGA gene families	Total VdRLs No.	VdRLs ID
R-IX	NB-ARC domain-containing disease resistance protein	9	VdRL07, VdRL08, VdRL11, VdRL12, VdRL13, VdRL15, VdRL16, VdRL18, VdRL26
R-II	Cysteine-rich RLK	4	VdRL05, VdRL06, VdRL21, VdRL23
R-V	Leucine-rich repeat protein kinase family protein	4	VdRL02, VdRL14, VdRL24, VdRL25
R-X	Receptor like protein	3	VdRL03, VdRL19, VdRL22
R-VII	Leucine-rich repeat transmembrane protein kinase	2	VdRL01, VdRL09
R-III	Disease resistance family protein_LRR family protein	1	VdRL10
R-VI	Leucine-rich repeat receptor-like protein kinase family protein	1	VdRL20
R-XI	TIR-NBS-LRR	1	VdRL04
Mixed	Mixed	1	VdRL17

Table S16 Verticillium wilt resistance QTL information of cotton

Marker name	Chr.	Position(cM)	Marker name	Chr.	Position(cM)
BNL1034	11	184.577	NAU1225	A13	59.8
BNL1122	16	87.8	NAU1230	D05	0.2
BNL1395	16	78.1	NAU2121	5	192.1
BNL1605	12/LG9	32.6	NAU2513	19	129.1
BNL1606	17	51.762	NAU2580	25	92.6
BNL1673	22	44.9	NAU2627	16	62.301
BNL2441	16	76.567	NAU2656	—	—
BNL2599	1	1.633	NAU2741	1	80.6
BNL2646	15	48.8	NAU2753	23	61.5
BNL2709	22	62.3	NAU2859	17	86.286
BNL2986	16	119.4	NAU2887	16	60.867
BNL3031	23	88.1	NAU2894	19	26.581
BNL3065	16	43.3	NAU2954	23	114.846
BNL3255	8	76.5	NAU3036	5	207.3
BNL3319	16	57.702	NAU3053	16	30.8
BNL3368	26	21.2	NAU3074	11	183.689
BNL3474	A02	170.6	NAU3200	—	—
BNL3537	26	67.1	NAU3201	24	39.3
BNL3590	2	57.4	NAU3212	5	66
BNL3646	20	3.479	NAU3279	16	129.1
BNL3649	21	10.8	NAU3287	—	—
BNL3867	12	154.4	NAU3481	21	136.1
BNL3971	2	88.7	NAU3499	8	65.3
BNL4069	19	36.8	NAU3574	20	58.598
BNL827	25	19.1	NAU3592	4	119.269
BNL830	15	128.7	NAU3607	5	17.1
CIR196	11	145.826	NAU3828	5	24.1
CIR364	19	66.663	NAU4912	26	0.0226
DPL0222	9	137.829	NAU5064	11	162.6
Gh268	—	—	NAU5075	—	—
Gh454	—	—	NAU5120	16	47.6
JESPR0001	19	123.567	NAU5233	3	108
JESPR101	17	71.031	NAU5273	5	86.1
JESPR232	A02	27.7	NAU5380	—	—
JESPR274	23	52.972	NAU5428	11	32.076
JESPR305	D03	83.9	NAU5463	—	—
MUCS219	D11.1	12.1	NAU5508	23	75.9
NAU1042	5	115.2	NAU751	16	130
NAU1043	7	167.5	NAU980	11	169.5
NAU1047	23	58.2	TMB1114	16	41.815
NAU1065	—	—			

* The markers in gray square cannot not be located on the *G. raimondii* genome. The Verticillium wilt resistance QTLs were reported in prrevious research [33-37].

Table S17 Primers used in this study

Name	Forward primer sequence	Reverse primer sequence	Remarks
Gorai.002G048900.1	5-GGAAAATAGAAAAAGTTCAGGACAT-3	5-ACATACAATTGCATAAAACCAGTTG-3	
Gorai.004G225800.1	5-ACAACATATCAGGAGACATTCCTTC-3	5-CTCGTTCAAAACCTTTCCATACAAC-3	
Gorai.004G248900.1	5-TTGATTACTTCGTTGATGGGGAG-3	5-GCTAGATTGGTTAGTTCTTTGGG-3	
Gorai.004G249900.1	5-GATAGACCAACCATGCGGGAA-3	5-TCATGGTTTAATCAAAATTATTTGCA-3	
Gorai.005G026700.1	5-TTGTTTCTTCAAAAGCTAGCCGAT-3	5-TCTCTCTCCCTCCAAAAATCCTGT-3	
Gorai.006G219400.1	5-AAAGAAAATGTGATACCGAAAGC-3	5-TCAACAACGGATCGAATAAACGA-3	
Gorai.007G045800.1	5-TGTGCAAGCCAAATCAGAAGAAA-3	5-ATACGAGAACCGCCATCAAAAGA-3	
Gorai.007G321700.1	5-ATGTGAAGGAAGTTTTGATTGTTC-3	5-ATTGTTTGATTTTCGCTCGTTTTG-3	qRT-PCR
Gorai.007G329900.1	5-ATTCATCGACTCCTTTTTGCTTC-3	5-CTGTCTTCCCCAATGTGTTACCA-3	
Gorai.007G364700.1	5-GAGAGTAGTTTGGGAAAGGCATT-3	5-AAGGAACCATTTTTGTTTTGATG-3	
Gorai.009G112500.1	5-CCCATTCTTTTTGTTCTTTCGTTT-3	5-CCTTTATTCATCTCCCAAGTTGCT-3	
Gorai.009G452000.1	5-CATAACTGATTGCTTTTGGTGC-3	5-AAGTCAAGGATTGTTCGGTGGC-3	
Gorai.011G052100.1	5-AGGATTGTATGAAGACAGGGAGA-3	5-TTGAAGTAAGATAAAGCGCAGGA-3	
Gorai.012G106100.1	5-GAAAGAAAGATTCAGACCTCCGT-3	5-AAACTTCCATCCTCAAAAACACC-3	
Gorai.012G117000.1	5-TCCCTAAGCTGGATCGCAGAA-3	5-CCAACATCCTACGACGCTCCT-3	
18S	5-CGGCTACCACATCCAAGGAA-3	5-TGTCACTACCTCCCCGTGTCA-3	Internal control
