# Supplementary material for: Genome-wide analysis of the gene families of resistance gene analogues in cotton and their response to Verticillium wilt
Source: BMC Plant Biol. 2015 Jun 19;15:148. doi: 10.1186/s12870-015-0508-3 (PMC4471920; doi:10.1186/s12870-015-0508-3)
Supplement: Additional file 2: — Supplementary figures. Figure S1. The statistics of RGA genes in G. raimondii chromosomes. The 11 families (R-I–R-XI) of RGA genes are cmarked in different colours. The X-axis represents the chromosomes of the G. raimondii genome (Chr01 to Chr13), and ‘Others’ represents the RGA genes that cannot be mapped to chromosomes at present. The Y-axis represents the number of genes. Figure S2. Homology clustering of RGA genes in the G. raimondii genome. Homology clustering was filtered using four conditions based on the match rates and identities among the RGA genes. Homology groups from HG01 to HG45 are arranged clockwise, the homology group intervals are differentiated by green and blue in series, according to the clustering conditions of match rate ≥ 33 % and identities ≥ 30 %. Figure S3. A sketch map of coding genes in Rgrcs. The coding genes in the Rgrcs are marked with a red line based on the physical map of the G. raimondii genome. For the genetic structures of the Rgrcs, RGA genes are represented by red squares and other genes are represented by black squares. Figure S4. Homology analysis of Rgrcs in the G. raimondii genome. (A) Homology analysis of the Rgrcs’ chimeric sequence. The chimeric sequence connected the Rgrc sequences in a series from Chr01 to Chr13 and was compared using the BlastN program (Version 2.2.23), ignoring self-matches and filtering out similarity blocks less than 3 kb in length. The forward-forward matches are marked with red lines, and the forward-reverse matches are marked with blue lines. (B) A statistical analysis of the similarity blocks among 26 Rgrcs. The lengths of the similarity blocks is greater than 3 kb. (C) Distribution of the identities of homology blocks. Figure S5. Cotton inoculated with V. dahliae. Two-week-old seedlings of the resistant cultivar G. barbadense cv. 7124 and the susceptible cultivar G. hirsutum cv. Jummian1 inoculated with the high virulence V991 defoliating strain of V. dahliae (5 × 10^6^ spores/ml). The phenotyp [file 12870_2015_508_MOESM2_ESM.pdf]

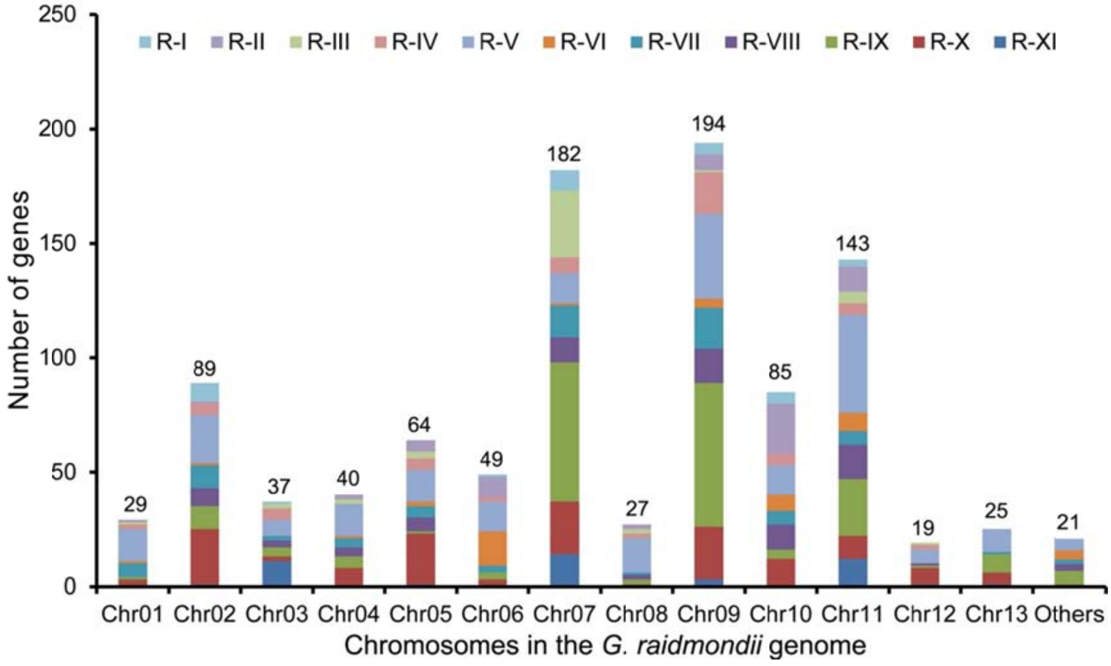

Figure S1 The statistics of RGA genes in *G. raimondii* chromosomes. The 11 families (R-I–R-XI) of RGA genes are marked in different colours. The X-axis represents the chromosomes of the *G. raimondii* genome (Chr01 to Chr13), and 'Others' represents the RGA genes that cannot be mapped to chromosomes at present. The Y-axis represents the number of genes.

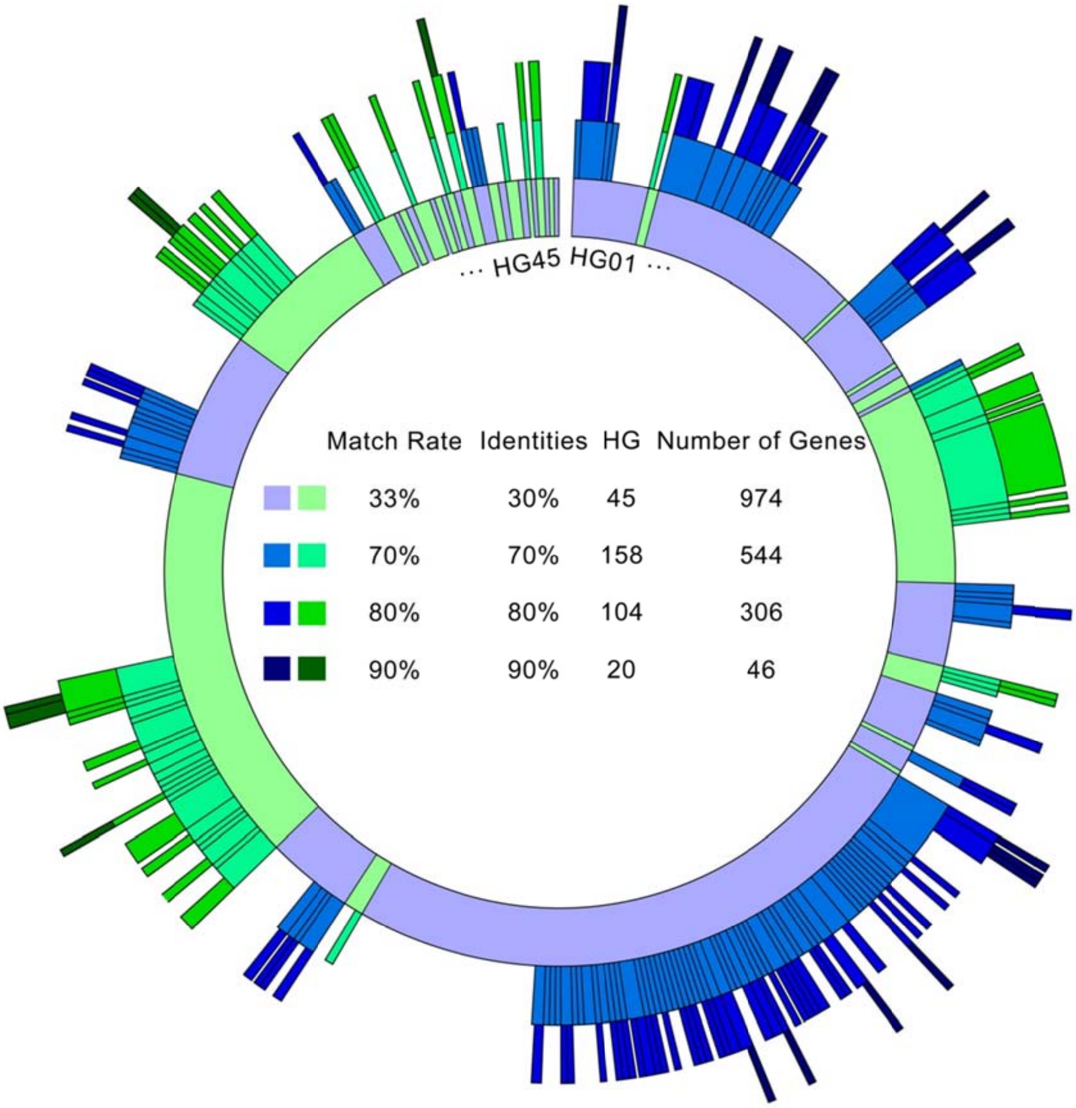

Figure S2 Homology clustering of RGA genes in the *G. raimondii* genome.

Homology clustering was filtered using four conditions based on the match rates and identities among the RGA genes. Homology groups from HG01 to HG45 are arranged clockwise, the homology group intervals are differentiated by green and blue in series, according to the clustering conditions of match rate ⩾ 33% and identities ⩾ 30%.

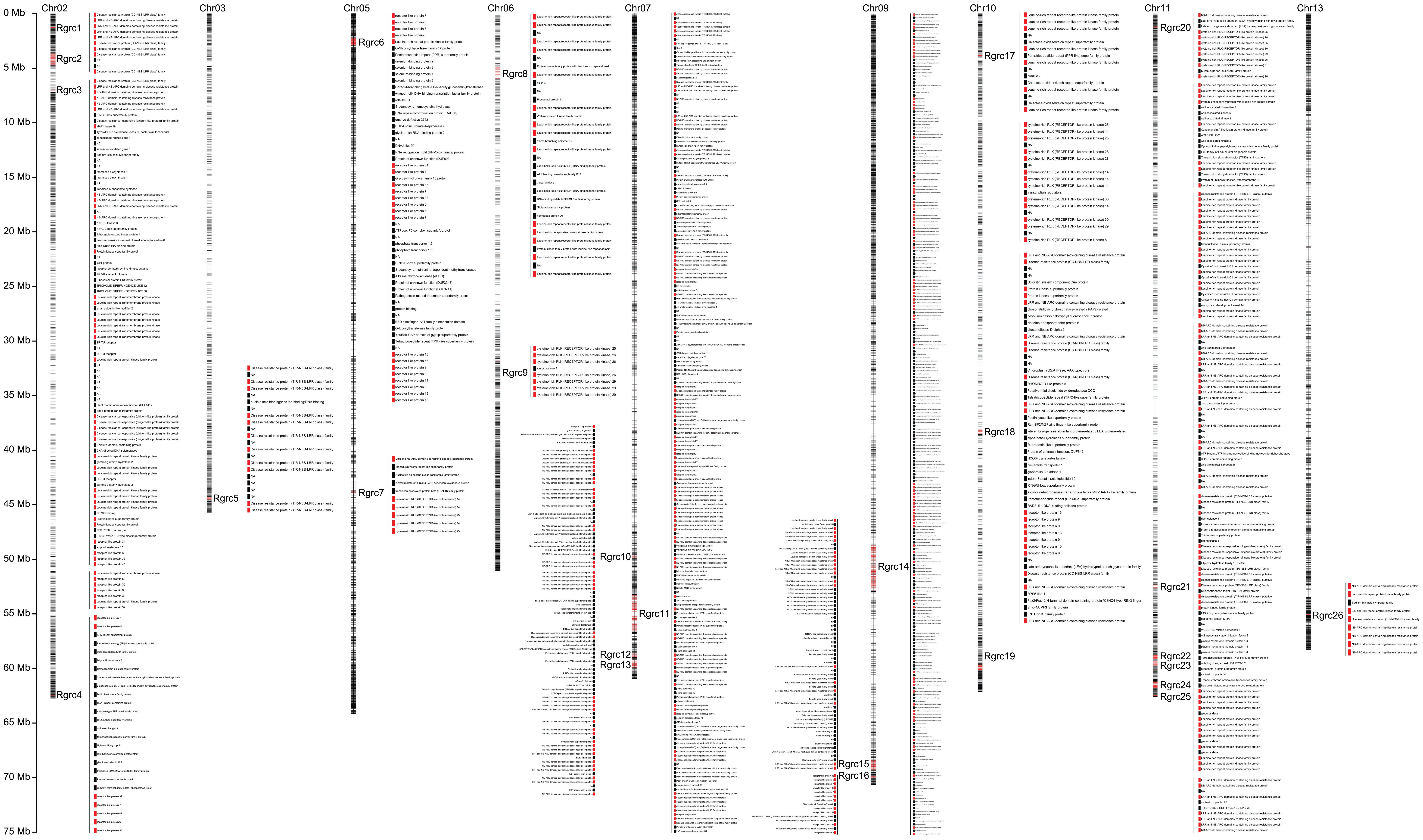

0 Mb
5 Mb
10 Mb
15 Mb
20 Mb
25 Mb
30 Mb
35 Mb
40 Mb
45 Mb
50 Mb
55 Mb
60 Mb
65 Mb
70 Mb
75 Mb
Chr02
Chr03
Chr05
Chr06
Chr07
Chr09
Chr10
Chr11
Chr13
Rgrc1
Rgrc2
Rgrc3
Rgrc4
Rgrc5
Rgrc6
Rgrc7
Rgrc8
Rgrc9
Rgrc10
Rgrc11
Rgrc12
Rgrc13
Rgrc14
Rgrc15
Rgrc16
Rgrc17
Rgrc18
Rgrc19
Rgrc20
Rgrc21
Rgrc22
Rgrc23
Rgrc24
Rgrc25
Rgrc26

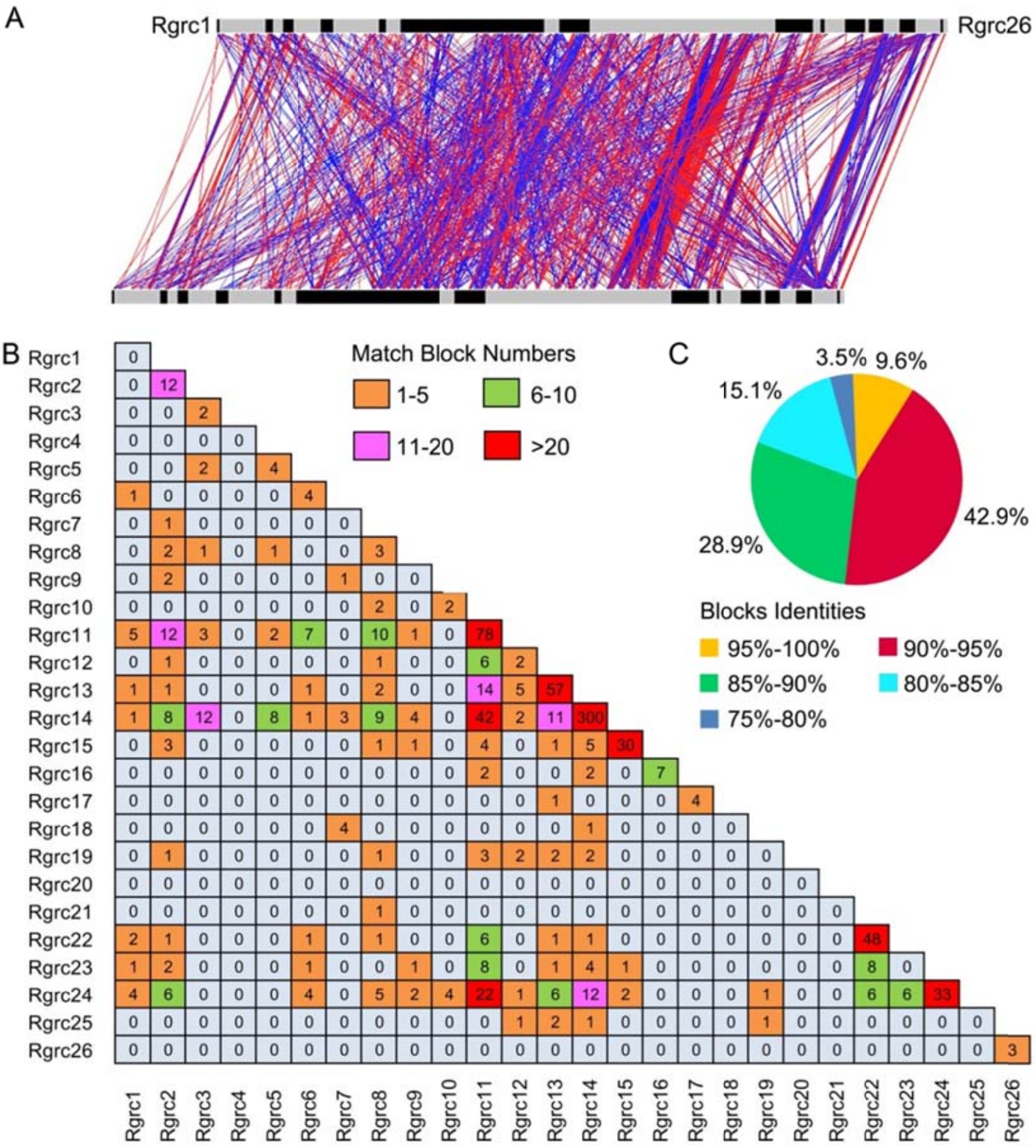

Figure S4 Homology analysis of Rgrcs in the *G. raimondii* genome. (A) Homology analysis of the Rgrcs' chimeric sequence. The chimeric sequence connected the Rgrc sequences in a series from Chr01 to Chr13 and was compared using the BlastN program (Version 2.2.23), ignoring self-matches and filtering out similarity blocks less than 3 kb in length. The forward-forward matches are marked with red lines, and the forward-reverse matches are marked with blue lines. **(B)** A statistical analysis of the similarity blocks among 26 Rgrcs. The lengths of the similarity blocks is greater than 3 kb. **(C)** Distribution of the identities of homology blocks

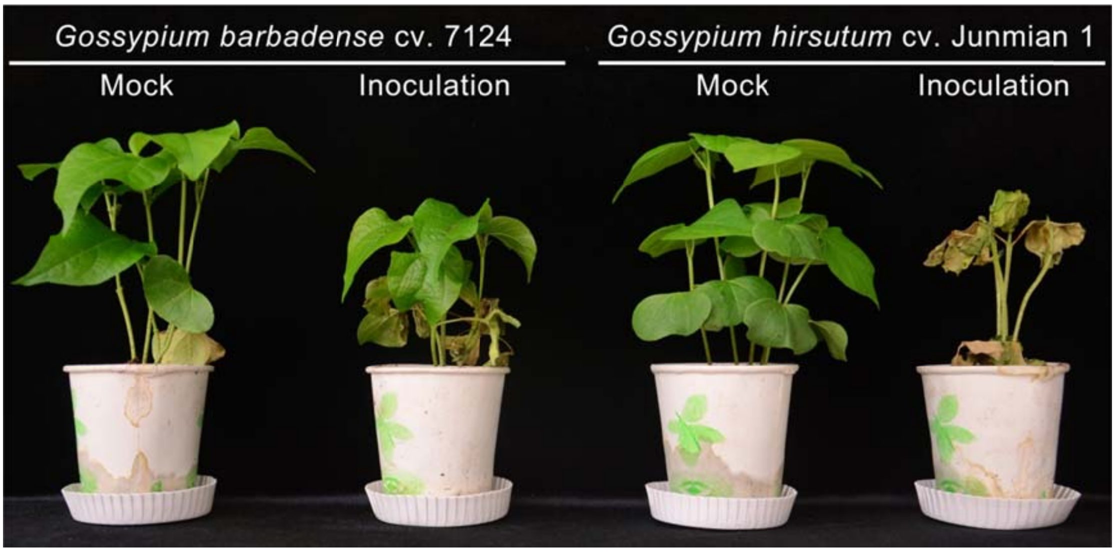

Figure S5 Cotton inoculated with *V. dahliae*. Two-week-old seedlings of the resistant cultivar *G. barbadense* cv. 7124 and the susceptible cultivar *G. hirsutum* cv. Jummian1 inoculated with the high virulence V991 defoliating strain of *V. dahliae* (5 × 10^6 spores/ml). The phenotypes were investigated three weeks after inoculation in this study.

.

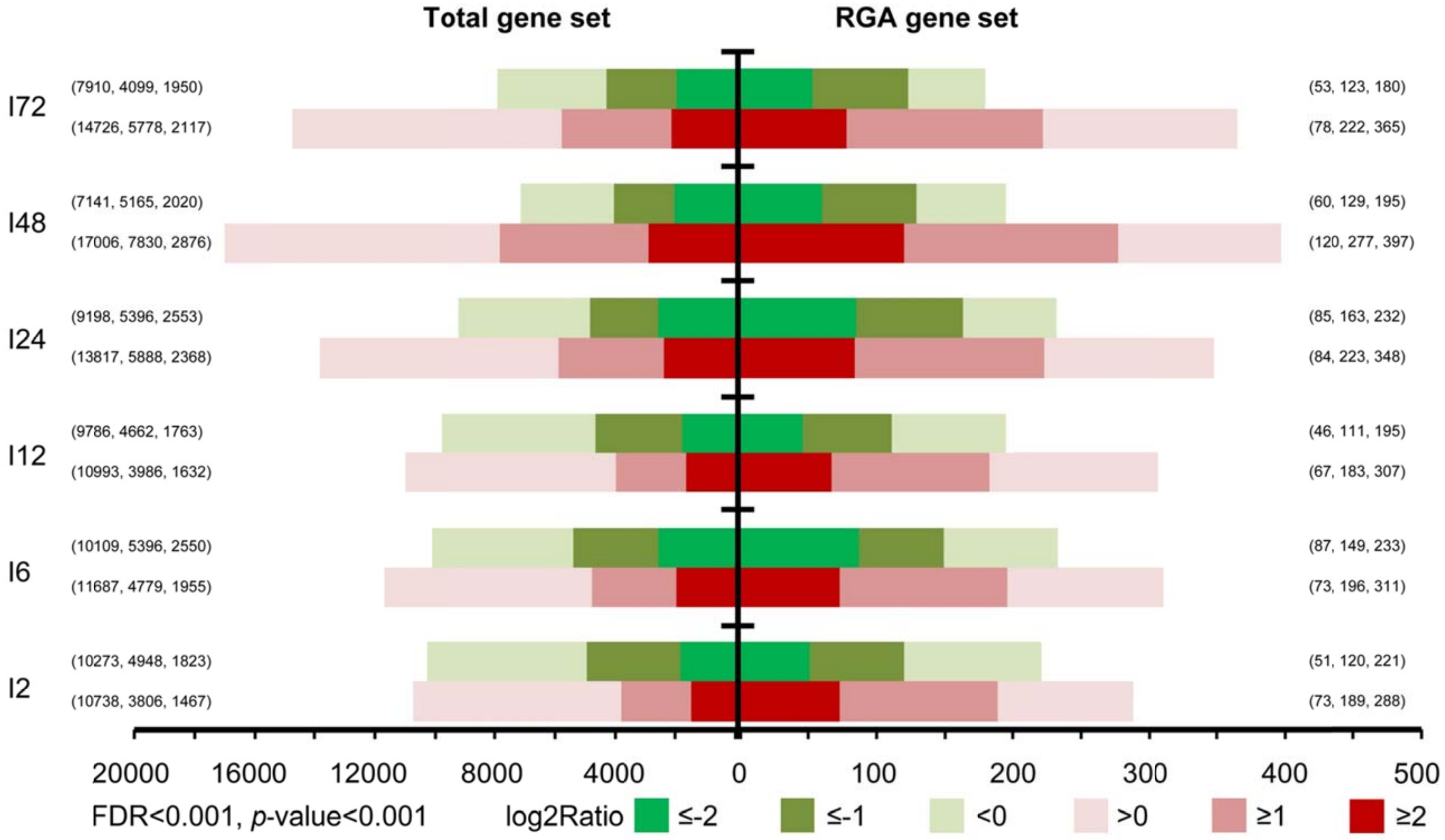

Figure S6 Statistical analysis of the DEGs in cotton inoculated with *V. dahliae*. The left side is the DEGs of all the genes in the *G. raimondii*

genome and the right side is the RGA gene set. The X-axis represents numbers of DEGs. ‘I2–I72’ represents the six inoculation time points (in hours). The numbers in the brackets from left to right represent the number of DEGs with more than a two-fold and a four-fold change, respectively, compared with mock-inoculated,. Red represents up-regulation and green represents down-regulation.

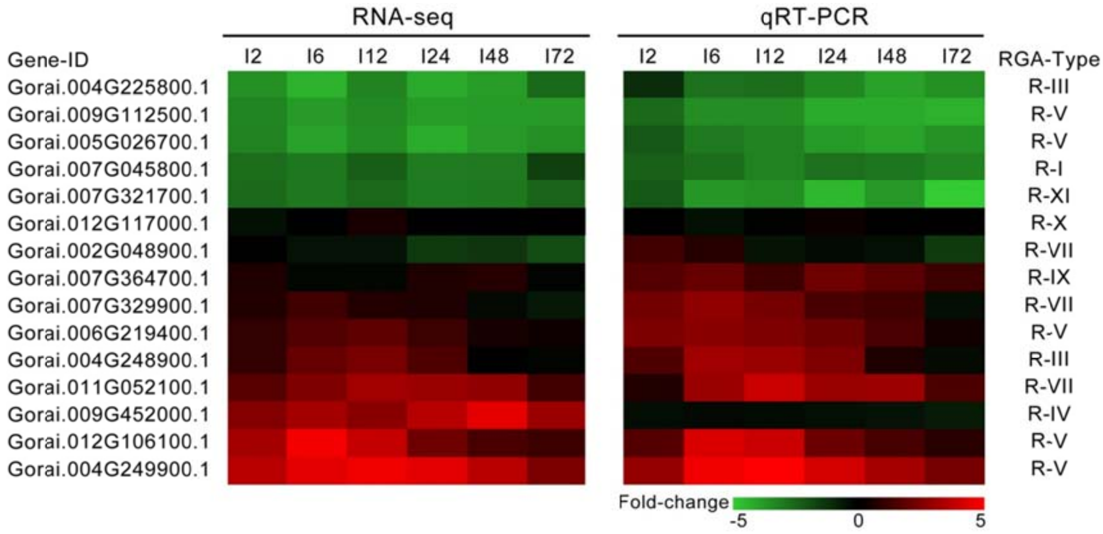

Figure S7 Differentially expressed RGA genes confirmed by qRT-PCR. In total, 15 differentially expressed RGA genes were randomly selected for qRT-PCR validation. The left side is the DEGs determined using RNA-seq and right side is the validation results using qRT-PCR.

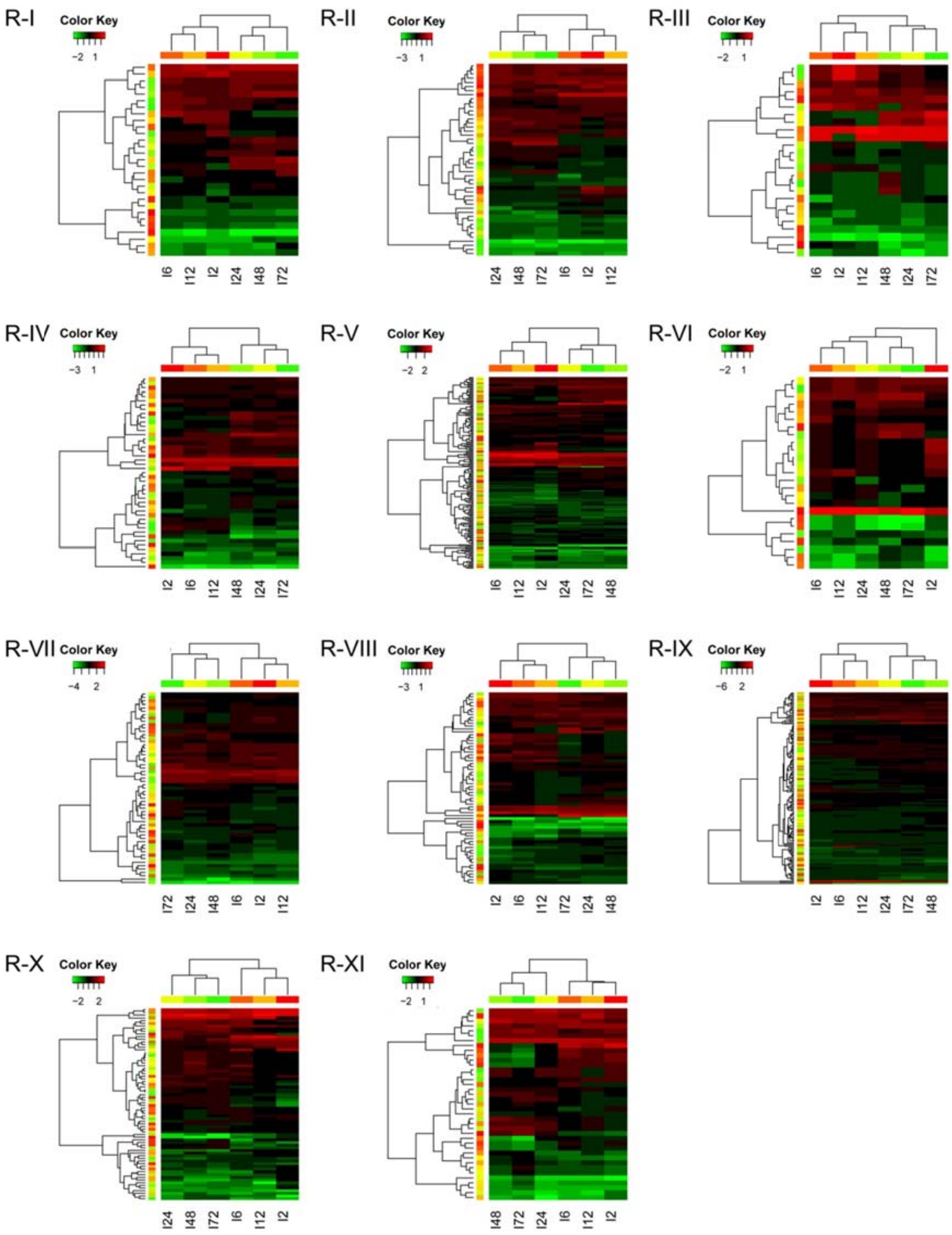

Figure S8 Expression pattern analysis of the response of 11 RGA gene families to *V. dahliae*. The filter conditions are FDR < 0.001 and $p < 0.001$. R-I–R-XI represents the 11 RGA gene families. 'I2–I72' represents the six inoculation time points.

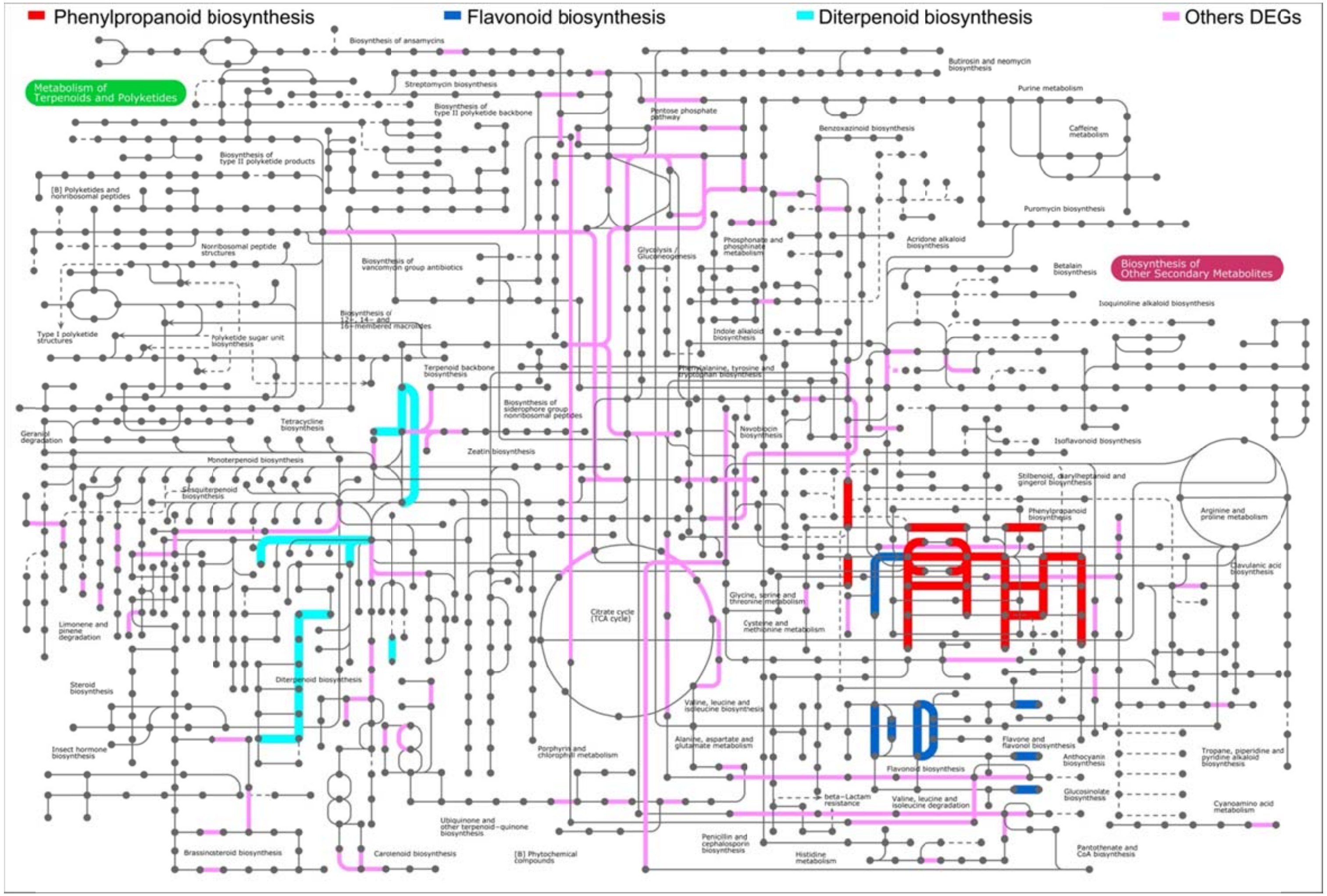

Figure S9 Phytoalexin biosynthesis pathway of *G. barbadense* inoculated with *V. dahliae*. The DEGs used for the metabolism pathway

analysis were screened by FDR < 0.001, $p < 0.001$, and $\log_2$Ratio $\geq |1.0|$ at all six inoculation time points. The thin lines represent the expression change of $\log_2$Ratio $\geq |1.0|$, and the thick lines represent the expression change of $\log_2$Ratio $\geq |2.0|$.

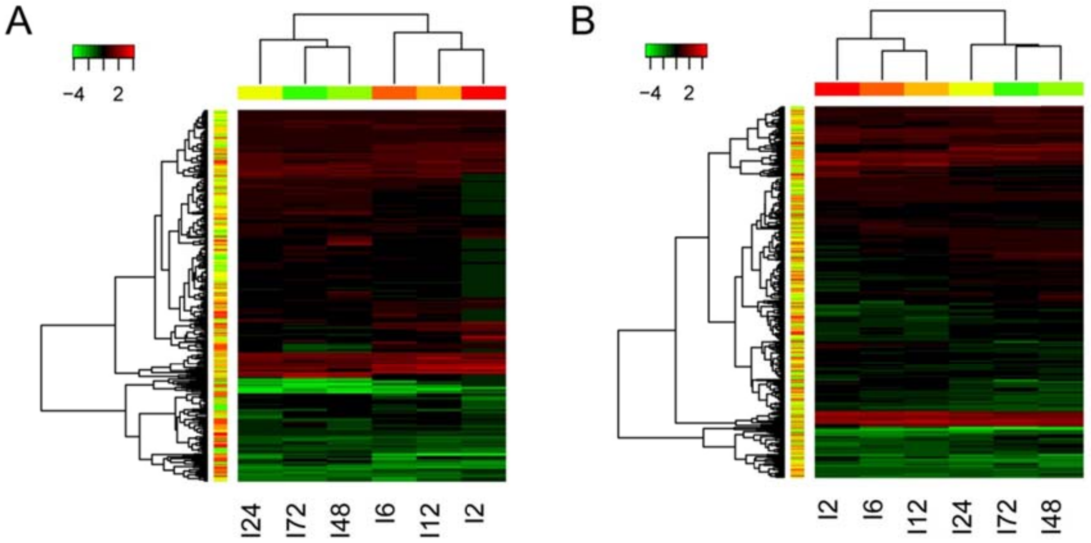

Figure S10 Clustering of DEGs encoded in Rgrcs. (A) The expression pattern analysis of RGA genes in Rgrcs. **(B)** The expression pattern analysis of other genes, not encoding RGA genes in Rgrcs. I2–I72' represents the six inoculation time points.
